# Update of the articular representation of the TLEM 2.0 musculoskeletal model

**DOI:** 10.1371/journal.pone.0358863

**Published:** 2026-09-21

**Authors:** Michele Conconi, Raphael Dumas, Nicola Sancisi

**Affiliations:** 1 Department of Industrial Engineering, University of Bologna, Bologna, Italy; 2 Univ Eiffel, Univ Lyon 1, LBMC UMR_T 9406, Lyon, France; Somaiya Vidyavihar University K J Somaiya College of Engineering, INDIA

## Abstract

To provide reliable results, musculoskeletal models must accurately represent human anatomy and be consistent with anatomical function. While considerable effort has been made to improve the muscular modeling, the same is often not true for the kinematic chain representing skeletal mobility. The aim of this work is therefore to take one of the most comprehensive musculoskeletal models, the TLEM 2.0, and improve its articular representation. Based on bone morphology, 3D articular kinematics of the tibio-femoral, patella-femoral, talo-tibial, and calcaneo-talar joints have been reconstructed by joint congruence maximization. Given these reference motions: the most isometric fibers for the main ligaments at the knee and the ankle have been identified; contact points and normals have been defined; the neutral configuration of the model has been defined. With respect to the original kinematic chain based on revolute joints, the one here proposed presents no joint distraction nor copenetration between the bones, together with a more physiological ligament elongation during joint motion, resulting in an articular representation more consistent with the TLEM 2.0 anatomy. These improvements substantially strengthen the anatomical fidelity of TLEM 2.0 and provide a robust foundation for next-generation MSK models. The updated dataset enables more reliable estimation of ligament and contact forces, supports the development of advanced rigid or deformable joint mechanisms, and offers a generalizable framework for subject-specific personalization whenever joint geometry is available. This represents a significant step toward MSK models capable of reliably capturing articular function under load.

## Introduction

Musculoskeletal (MSK) models are now widely used in biomechanics for quantitative diagnostics, functional evaluation of prostheses and implants, and the development of data-driven gait rehabilitation protocols [1]. Despite their growing adoption, their translation into clinical practice remains limited. This gap may stem from the simplifications commonly applied in modelling MSK structures, the challenges associated with adapting models to individual subjects, and the anatomical inconsistencies across different levels of MSK analysis, from inverse kinematics, to inverse dynamics and static optimization, up to joint reaction computation [2–4].

Among the many available implementations, the TLEM 2.0 [5] sought to mitigate the common limitations of MSK models, providing the highest level of detail currently available for muscular geometrical information, including muscle-tendon lines of action and wrapping surfaces, carefully derived from imaging and direct palpation of a donor. However, the model is not equally detailed in terms of skeletal kinematics chain. Indeed, the main articulations in the lower limb, namely the tibio-femoral, patello-femoral, talo-tibial, and calcaneo-talar joints, are represented simply through revolute joints based on axes calculated based on a cylindrical fit through the trajectory of points of the distal bone with respect to the proximal bone. While revolute joints may provide an acceptable approximation when computing muscle lever arms, they are not sufficient when the MSK model aims at analysing articular behaviour. In this case, more advanced models are required, capable of taking into account the whole three-dimensional (3D) kinematics and being consistent with articular constraints. Among these models, there are, for instance, the coupling between degrees of freedom (DOF), rigid or deformable mechanisms with contact surfaces and ligaments, contact point trajectories [4,6–13].

To provide a more complete and consistent MSK model, this work aims to expand the TLEM 2.0 dataset by adding a detailed representation of the knee and ankle articulations, including ligaments, contacts, and 3D joint kinematics. This is achieved by exploiting previously developed methods capable of predicting the individual joint motion and identifying ligament isometric fibers from articular morphology [14,15]. The definition of the TLEM 2.0 hip joint (spherical centre and ligaments) was not updated in this study.

In particular:

- tibio-femoral, patello-femoral, talo-tibial, and calcaneo-talar kinematics will be reconstructed using joint congruence maximization, estimating cartilage as a constant thickness layer, insisting on subchondral surfaces.
- Based on this motion, the neutral leg configuration will be defined and an updated version of the mean helical axes will be provided.
- Contact points and normals associated with the maximum congruence motion will be computed for each articulation.
- Finally, based on atlas, the insertions of the main ligaments at the knee and ankle will be updated or defined, and within them, the most isometric fiber will be identified for each ligament based on the maximum congruence motion.

In the end, an atomically consistent set of articular kinematics and geometry (both obtained from contacts and ligaments) will be provided for all lower-limb joints, to be used to implement a MSK capable of capturing articular and ligament contributions to the joint reaction forces and moments.

## Materials and methods

### Cartilage estimation

**Fig 1:**
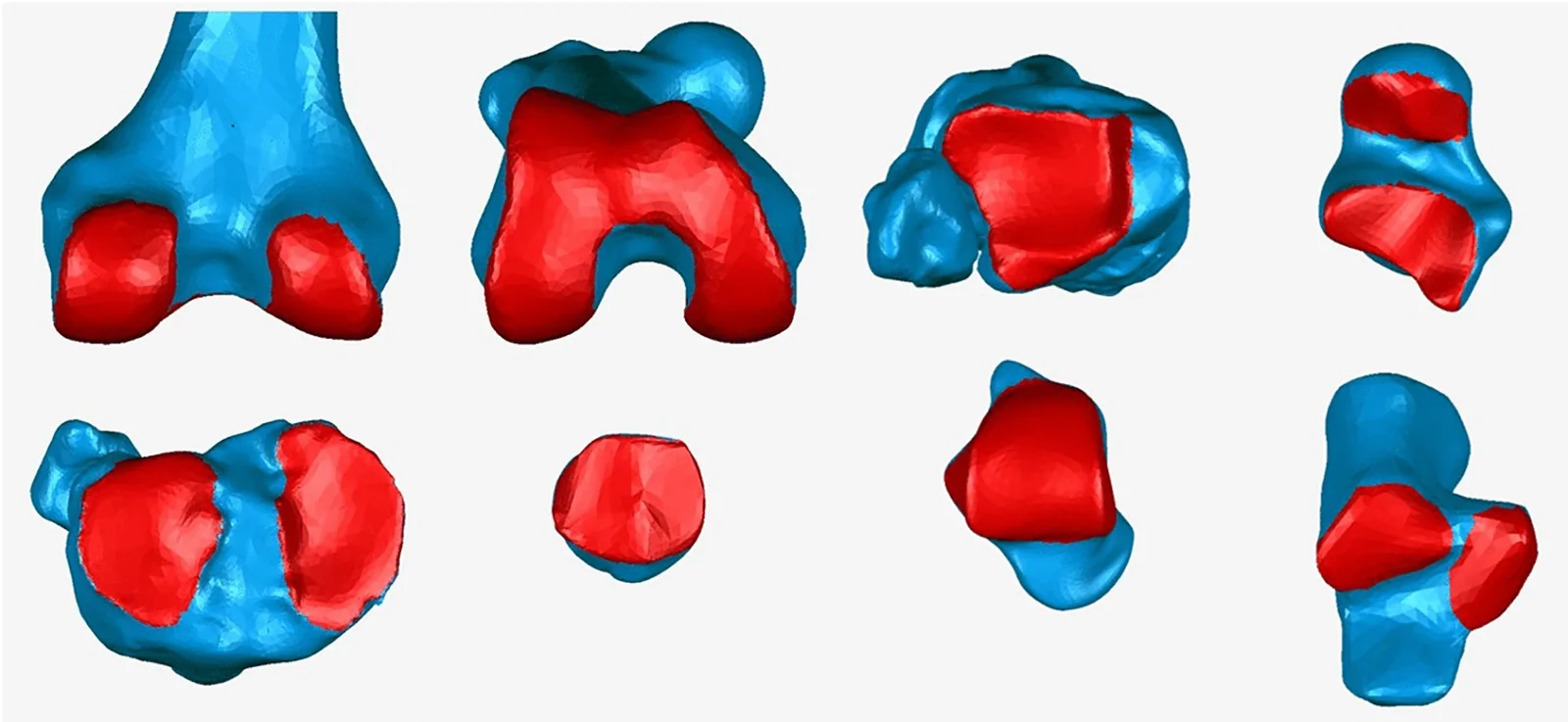
Selection of the subchondral surfaces (in red) for the various bones and joints.

Starting from the bone models segmented from CT images and available in the TLEM 2.0 dataset [5], subchondral surfaces were manually selected for each articulating bone of the four considered articulations (tibio-femoral, patello-femoral, talo-tibial, and calcaneo-talar) using Geomagic Studio 12.0 (Fig 1). These selections were then offset within the same software to resemble a constant cartilage layer, whose thickness was determined by averaging data from the literature [16–20]. The chosen values are reported in Table 1.

**Table 1:** Chosen values for the definition of constant thickness cartilage layers on each articulation.

	Femur trochlea and condyles	Tibia plateaus	Patella	Tibia undersurface	Talar dome	Subtalar surface	Calcaneus
thickness	2 mm [16]	2.2 mm [16]	2.7 mm [17] [18]	1.3 mm [16]	1.2 mm [16]	1.3 mm [19] [20]	1 mm [19] [20]

### Computation of joint kinematics

Once the articular surfaces were reconstructed, the joint kinematics were determined via congruence maximization [14,15,21,22]. The method assumes that articular shape results from an optimization process that minimizes peak contact pressure, or, in other words, maximizes joint congruence [23]. Articular motion is thus obtained as the envelope of subsequent maximum congruence positions and orientations (poses).

To this purpose, reference systems have been defined to describe articular kinematics, with the goal of minimizing secondary motion components and simplifying numerical optimization. For the femur and tibia at the knee, we adopted the method proposed in [24]. For the patella, we adopt what proposed in [25]. For the tibia, talus, and calcaneus at the ankle, we adopted the approach proposed in [26].

The motion of the tibia relative to the femur was parametrized using the standardized Cardanic sequence [27], thus rotating the tibia about its axes in the sequence z-x-y so that the final rotational matrix will result in $=R_{z}R_{x}R_{y}$. Translations were described as the positions of the tibia reference system’s origin with respect to the femur reference system. The same approach was used to describe the pose of the patella with respect to the femur. At the ankle, we adopted a similar approach; however, we changed the rotation order to minimize the secondary components of motion between the talus and the tibia, and between the calcaneus and the talus, following what was proposed in [28,29]. In this case, the sequence of rotation about the axes of the distal bone is z-y-x, so that the final rotational matrix will result in $R=R_{z}R_{y}R_{x}$.

All the joints were considered with one DOF, so that one coordinate can be freely assigned while the secondary ones are determined by maximizing joint congruence. At the tibio-femoral and talo-tibial joint, the independent coordinate is the flexion (rotation about the z axis), while at the calcaneo-talar joint, the free coordinate is the rotation about the x axis, corresponding to the foot inversion [30]. The patello-femoral pose was considered dependent on the tibio-femoral flexion angle: to determine its trajectory, the tibia was positioned at the pose found by maximizing congruence for the considered flexion angle. Patello-femoral congruence was then maximized while guaranteeing a constant length of the patellar tendon [21], whose insertions were provided in the TLEM 2.0 dataset, while the resting length was determined as the insertion distance at the CT-scan pose provided in the database. As the patella does not fully articulate with the femur in the first part of the knee flexion, the patello-femoral motion cannot be obtained via congruence maximization on the whole flexion range. To ensure that congruence calculation takes place when the patella is fully engaged with the trochlea, patello-femoral congruence maximization was computed only for flexion values greater than 40°. The initial part of the patellar trajectory was described as a rotation about and translation along the single finite helical axis that connects the patella pose measured from the CT-scan to the first pose computed by congruence maximization at 40° of flexion: given the two rototranslational matrices, the finite axis of motion can be uniquely determined thanks to the Mozzi-Chasles’ theorem [31].

The range of motion (ROM) for each articulation was selected from the literature to ensure that the extreme poses would not result in abnormal contact, particularly at the ankle (Table 2). Computation was performed by varying the independent DOF of 1° within the chosen ROM.

**Table 2:** Chosen independent degrees-of-freedom (DOF) and corresponding range-of-motion (ROM) for the computation of maximum congruence kinematics at the considered articulations.

	tibio-femoral -patello-femoral	talo-tibial	calcaneo-talar
Independent DOF	Knee flexion-extension	Ankle flexion-extension	Foot supination-pronation
ROM	[−5, 120]	[−10, 23]	[−10, 14]

Following the modification proposed in [22], the control volumes determining articular congruence were defined by offsetting solely the articular surfaces. For this purpose, the same process and selection used to define cartilage layers were employed. It is worth noting that this differs from what was proposed for the knee in a previous study [15], in which a control volume was constructed assuming the menisci were fixed to the tibia plateaus. In this case, however, no information on these articular structures was available in TLEM 2.0.

At the end of the computation, the kinematics of all the joints was converted to the reference systems employed in the TLEM 2.0 dataset [5]. Furthermore, to make the results independent of the chosen pose parametrization, the kinematics is provided as rototranslational matrices in the supplementary material.

### Definition of the neutral posture

The lower-limb neutral posture derived from the revolute joints of the TLEM 2.0 dataset resulted in an unusual orientation of various leg segments (Fig 6). Moreover, the configuration of the foot bones derived from the CT images shows a deviation from normal foot posture. However, a solid definition of a neutral lower limb is necessary to accurately scale the model across different subjects. With the goal to extend the consistency of the model also to this aspect, we redefine the lower-limb neutral posture as follows:

- The calcaneo-talar neutral pose was chosen as the one that exhibits the maximum congruence over the whole range of motion trajectory.
- Given the calcaneo-talar joint in this relative pose, the mid- and the forefoot mesh was taken from the original TLEM 2.0 database and considered as a single rigid body: the configuration of this cluster was manually modified to eliminate bone copenetration and to result in a global foot posture compatible with neutral foot contacting the ground, so that the naviculo-talar and the cuboid-calcaneal articulations are in a physiological configuration.
- Once the neutral foot was defined, the tibio-talar neutral pose was then identified as the one for which the normal to the ground, defined as the plane through the most distal point of the calcaneus, the first and the fifth metatarsal heads, forms the smallest angle with the y axis of the tibia reference system.
- Finally, the knee angle was chosen so that the angle between the y axis of the femur and the tibia reference system was minimal. The neutral pose of the patella relative to the femur was kept from the original dataset (CT-scan pose).

Given the articular motions, the envelopes of instantaneous helical axes (IHA) were computed following the approach proposed in [32] and reported in the supplementary material of [30] for the sake of completeness. From here, the mean helical axis (MHA) corresponding to each IHA envelope was also computed, following the approach reported in [33] and in the supplementary material of [30]. The analysis was performed for all the articulations twice, considering both the motion of the distal bone relative to the proximal one and vice versa. Indeed, the IHA envelope and consequently the MHA depend on the observer, even if the relative motion remains the same, and therefore the two approaches will provide two different MHAs, one per bone.

### Contact points and normals

Contact analysis was performed by dividing articular surfaces into medial and lateral regions, except for the calcaneo-talar one, which was separated into an anterior and a posterior region. To evaluate point-to-surface distances, a distance map was computed for each bone’s mesh, with simulated cartilage, as in [31].

For each articulation and region, contact areas were determined as the portion of the articular surfaces whose distance was below a 2 mm threshold from the mating surfaces. The single point of contact was computed as the centroid of the contact areas, then projected on the articular surface itself. In this way, a pair of contact points was determined, one on each articulating bone and contact region, for any considered pose.

Using the same mathematical tools, a pair of contact normals, one for each articular surface, was computed as the average of the mesh normals in the corresponding contact area, weighting each normal contribution by k=(t−d), where *t* is the threshold value and *d* is the dis*t*ance of the center of the considered mesh facet from the mating surface.

### Redefinition of ligaments

TLEM 2.0 origins and insertions are provided as single points and only for four principal knee ligaments, namely anterior (ACL, two bundles) and posterior (PCL, two bundles) cruciate, medial (MCL) and lateral (LCL) collateral ligaments. These ligament attachments are, however, not consistent with the provided axes of motion, as they result in ligaments that are far from being isometric during knee passive flexion [33–35], also resulting in unphysiological elongations (see Figs 8 and 9).

For this reason, the origins and insertions of the ACL, PCL, MCL, and LCL were remapped onto the bone meshes by manually selecting the areas based on anatomical atlases, thus resulting in two clouds of points for each ligament instead of a pair of points. With this process, the origin and insertion of the anterior lateral ligament (ALL) and the anterior (MCLDa) and posterior (MCLDp) bundles of the deep fascia of MCL in the knee were also added to the model. To introduce similar information for the patello-femoral and the ankle joint, the origin and insertions of the superior medial (LPFLs), inferior medial (LPFLi), superior lateral (LPFLs), and inferior lateral (LPFLi) patello-femoral ligaments, as well as tibio-calcaneal (TICAL) and fibulo-calcaneal (FICAL) ligaments, were also identified.

Finally, to provide a single fiber representation of the ligaments consistent with the maximum congruence motion, we numerically searched the origin and insertion point clouds of each ligament to identify the two points showing the most isometric behavior during natural motion, where the isometry is defined as:


$$\begin{array}{c} \Delta L=\frac{L_{max}-L_{min}}{L_{max}} \end{array}$$

Where L is the distance between the two points (i.e., the fiber length). The isometry of the tibio-femoral, patello-femoral, and ankle ligaments was defined based on the computed kinematics. While studying the ankle, the foot (and the calcaneus in particular) was kept in its neutral pose.

## Results

### Joint kinematics

**Fig 2:**
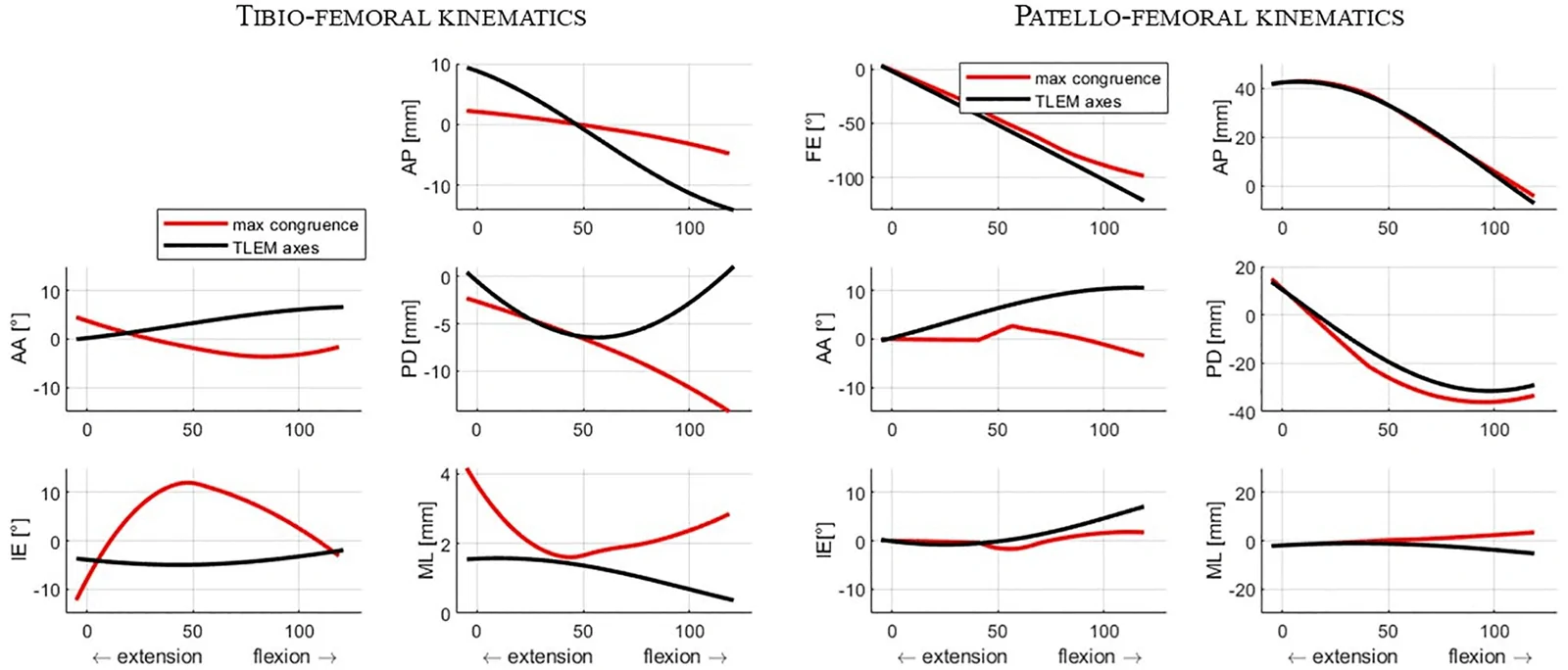
Comparison between revolute-based kinematics from TLEM 2.0 (black) and maximum congruence (red) motion for the tibio-femoral (left) and patello-femoral (right) joints.

**Fig 3:**
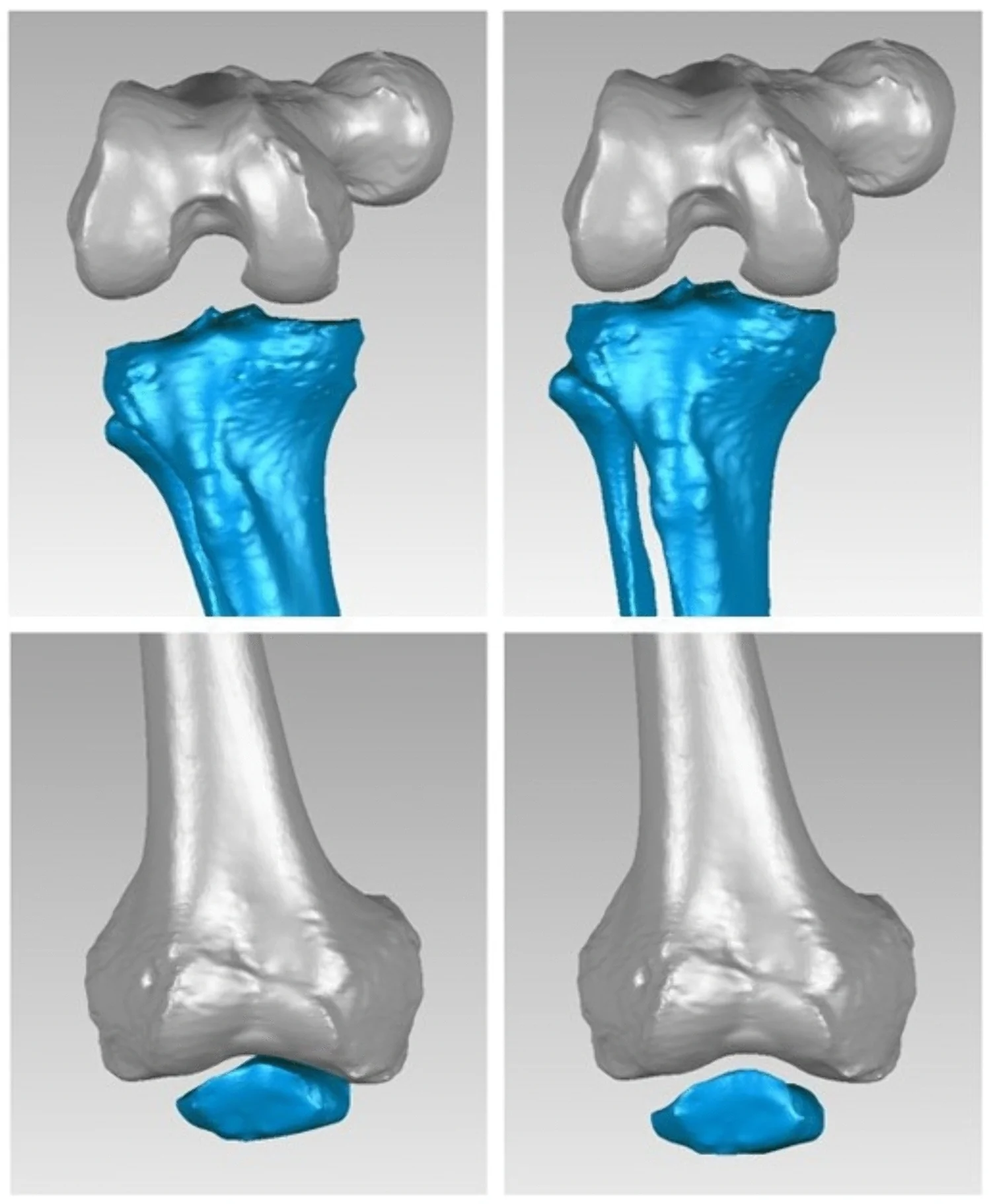
Visualization of the tibio-femoral (up) and patello-femoral (down) pose at 90° of flexion for the revolute-based (left) and the maximum congruence (right) kinematics. On the left, it is possible to observe joint distraction (top) and bone-to-bone co-penetration (bottom), while on the right, a uniform articular gap is preserved.

In Fig 2, the knee joint kinematics resulting from both the original revolute pairs in TLEM 2.0 and the maximum congruence approach are reported. The two models yield sensibly different kinematics at the tibio-femoral joint, particularly for the internal-external (IE) rotation; at the patello-femoral joints, data are in good agreement, with some differences at the abduction-adduction (AA). In general, maximum congruence motion is in good agreement with the previous literature [15,36,37]. Fig 3 shows the same comparisons by visualizing the 3D pose for the tibio-femoral and patello-femoral joint at 90° of flexion, where it is possible to observe considerable joint distraction and bone copenetration associated with the revolute-based kinematics, while the maximum congruence motion respects the physiological bone-to-bone articular gaps.

**Fig 4:**
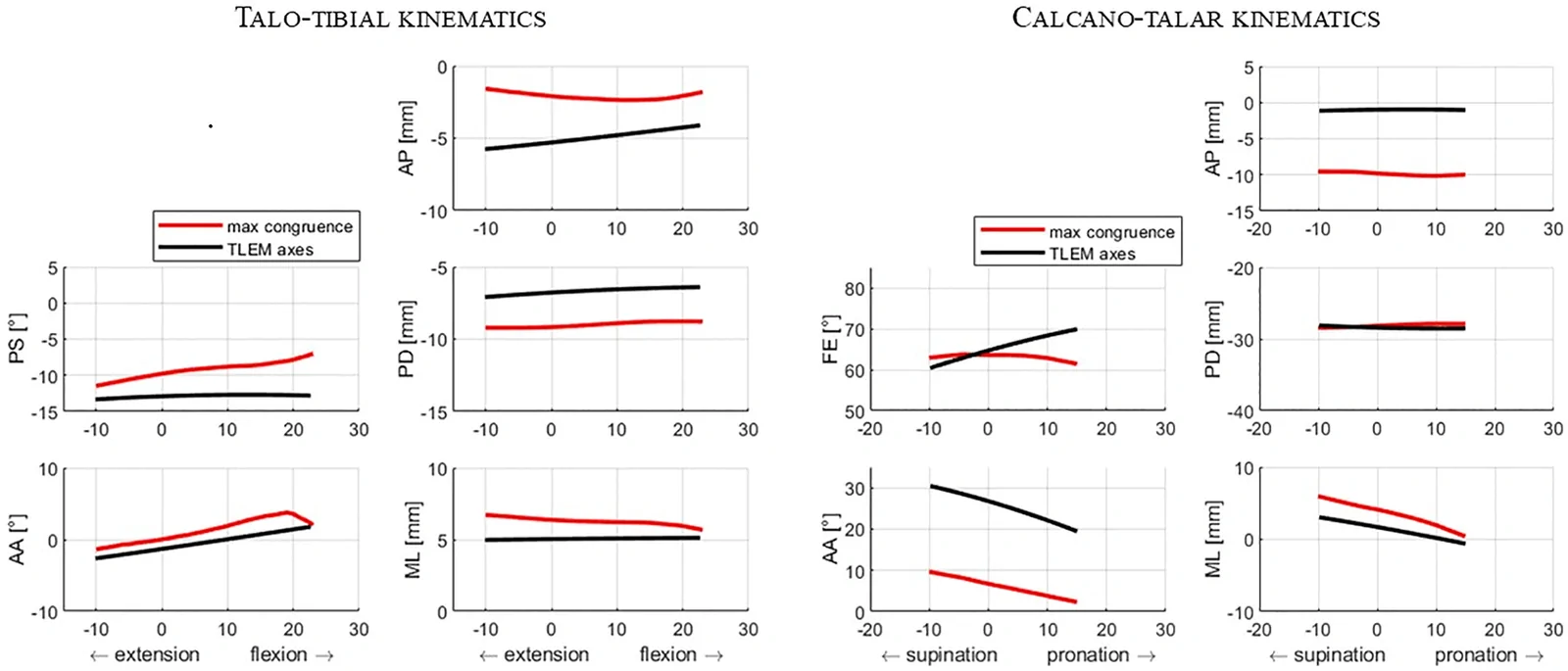
Comparison between revolute-based kinematics from TLEM 2.0 (black) and maximum congruence (red) motion for the talo-tibial and calcaneo-talar joints.

**Fig 5:**
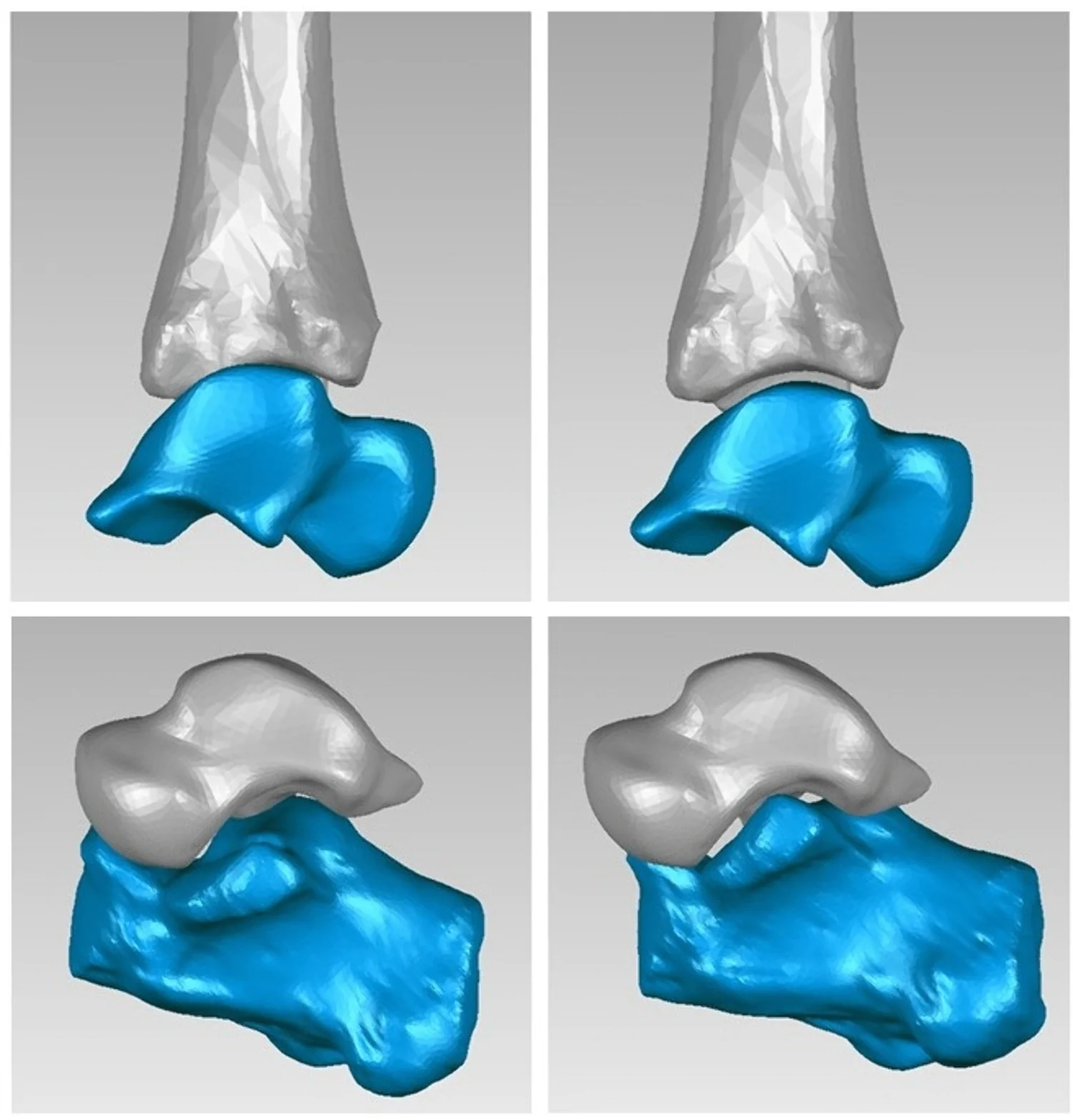
Visualization of the talo-tibial (up) and calcaneo-talar (down) joint neutral pose for the revolute-based (left) and the maximum congruence (right) kinematics. On the left, it is possible to observe bone-to-bone co-penetration at both the articulations, while on the right, a uniform articular gap is preserved.

Similarly, in Fig 4, the computed talo-tibial and calcaneo-talar kinematics resulting from the two modeling approaches are reported. Again, some non-negligible differences are observable, with the revolute-based kinematics resulting in unphysiological bone-to-bone copenetration (Fig 5), while the maximum congruence model preserves a uniform articular gap and is in better agreement with previous literature [28].

An anatomically consistent and reliable quantification of the articular kinematics resulting from congruence maximization is reported for all the joints in the supplementary material. Each relative pose is provided as a rototranslational matrix, written line-by-line in a single 1x16 vector, using the reference system from the original TLEM 2.0 dataset.

The associated MHAs are also available in the supplementary materials, reported as an unit vector and a specific point on the axis in the reference system provided in the original TLEM 2.0 dataset.

### Neutral posture

**Fig 6:**
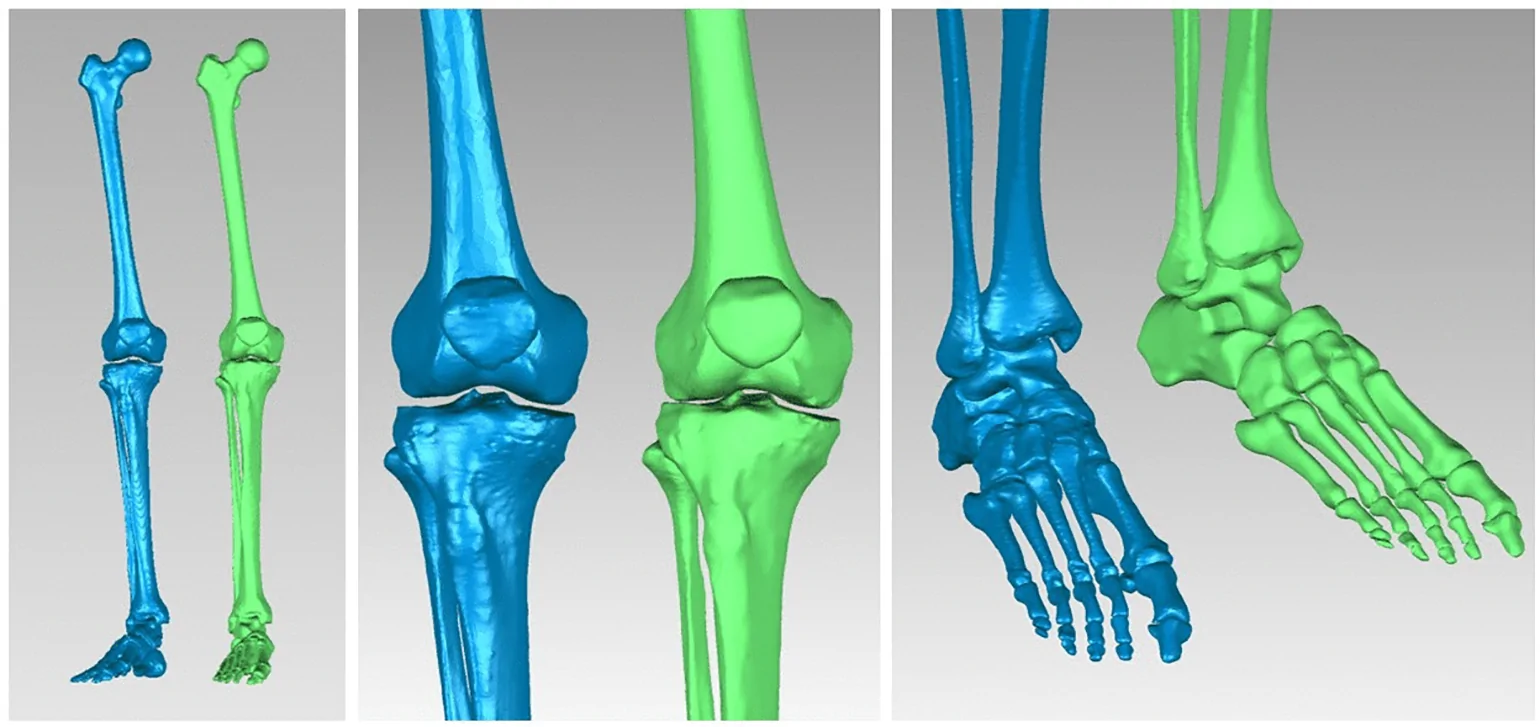
Original (green) and corrected (blue) neutral posture of the leg.

In Fig 6, the original and updated neutral leg postures are shown. At the knee, the updated pose results in a more uniform articular gap, whereas the previous natural pose produces a moderate valgus knee with a reduced lateral gap. The original alignment of the foot results in copenetration between talus and calcaneus and a disarticulated talo-navicular joint, while the updated pose respects the articular anatomy of the hindfoot.

The transformation matrices representing the updated posture are provided in the supplementary material, in the homonymous folder, where the neutral poses of each joint are expressed using the reference system provided in the original TLEM 2.0 dataset.

### Contact points and normals

**Fig 7:**
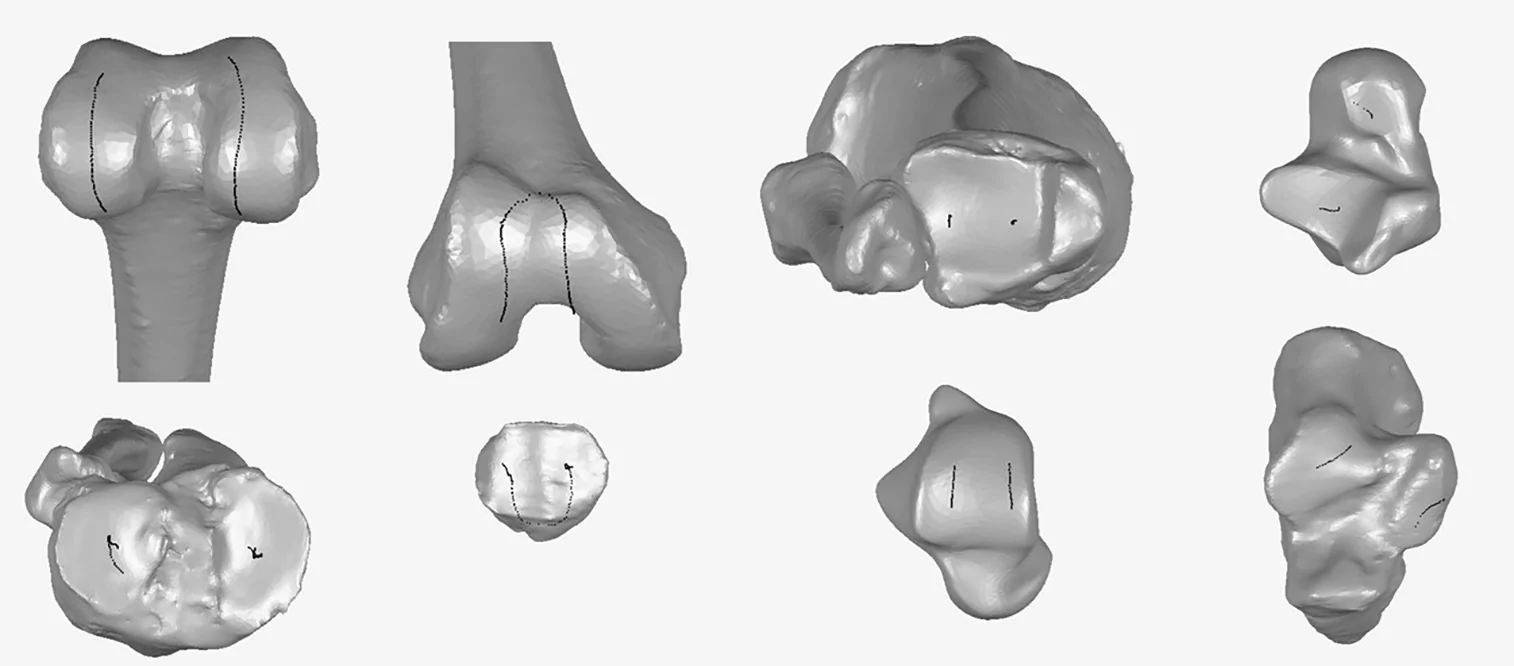
From left to right: trajectory of contact points on the tibio-femoral, patello-femoral, talo-tibial, and calcaneo-talar articulations.

In Fig 7, the contact point trajectories for the various considered articulations associated with the computed motion are reported. Results are in agreement with what was previously reported in the literature on active kinematics: at the knee, the medial contact on the tibia plateau remains almost stationary in the center of the contact area, while on the lateral plateau, the contact moves posteriorly while the knee flexes, in agreement with the medial pivot representation of this articulation. On the femoral counterpart, the contact points move along the condyles following the flexion of the knee. A similar behavior is observable for the femoral part of the patello-femoral articulation, where contact points move along the trochlea groove. On the patella, contacts initiate distally and soon stabilize in the center of the articular surfaces [38].

At the tibio-talar joint, it is possible to observe an antero-posterior motion of the talar contact points with the ankle plantarflexion, while the calcaneal contact point on the posterior facet moves posteriorly and laterally while the calcaneus supinates [39].

Contact points and normals are available in the supplementary material as matrices, with each line containing the coordinates of the corresponding contact points or the orientation of the corresponding contact normals; each matrix has as many rows as the poses of the corresponding motion. Data are reported in the reference frame (provided in the original TLEM 2.0 dataset) of both articulating bodies.

### Ligament elongation

**Fig 8:**
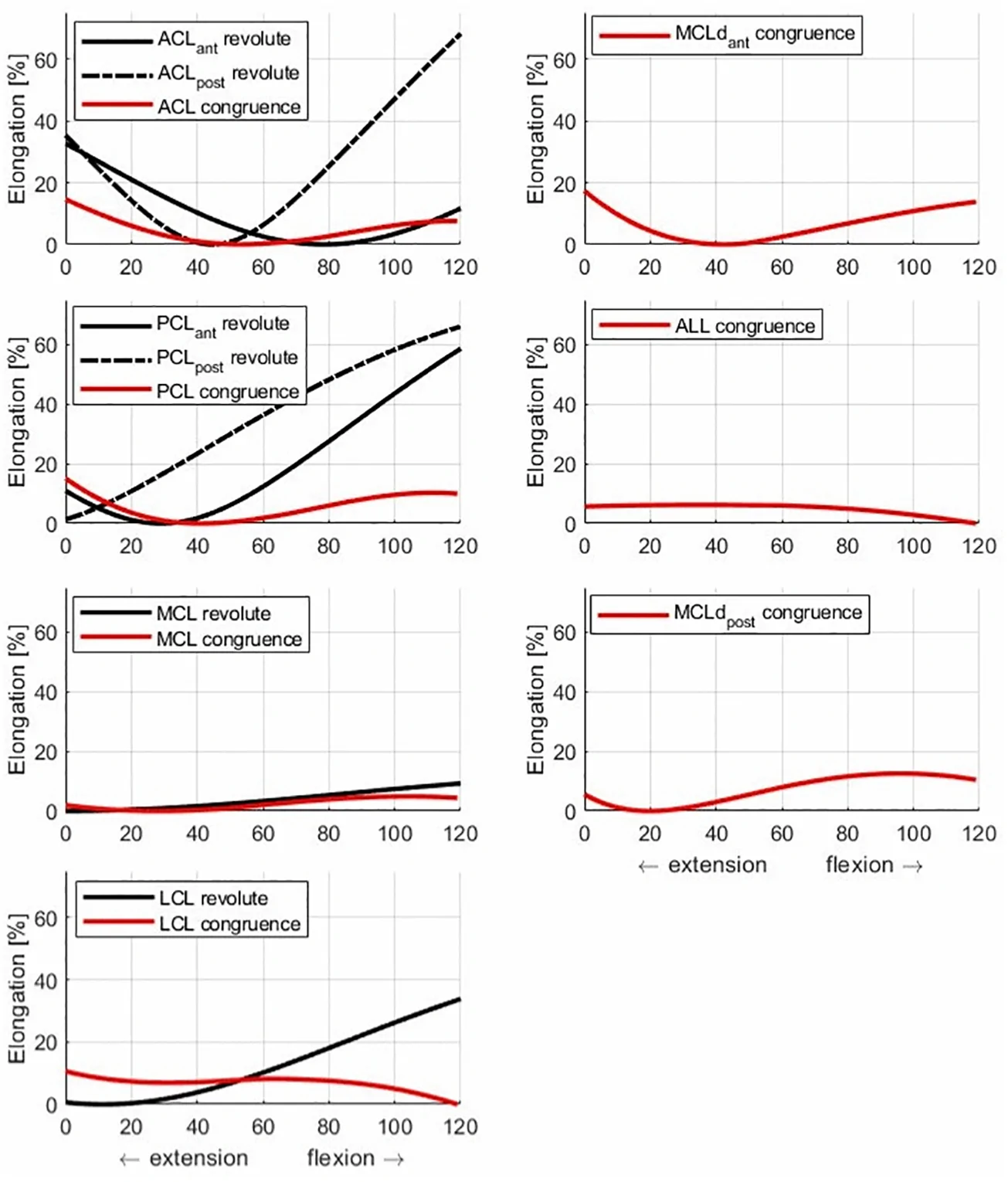
Percentage ligament elongation for the revolute-based (black) and maximum congruence (red) motion at the tibio-femoral joints.

In Fig 8, the percentual length elongation of the original knee ligament, in association with the revolute-based motion, is compared with that of the most isometric ligament, in association with the maximum congruence motion. The originally provided fibers clearly result in unphysiological tensioning, particularly at the ACL and PCL, where elongation exceeds 40%. The maximum congruence motion allows for the identification of nearly isometric fibers for all the considered ligaments [33,34].

**Fig 9:**
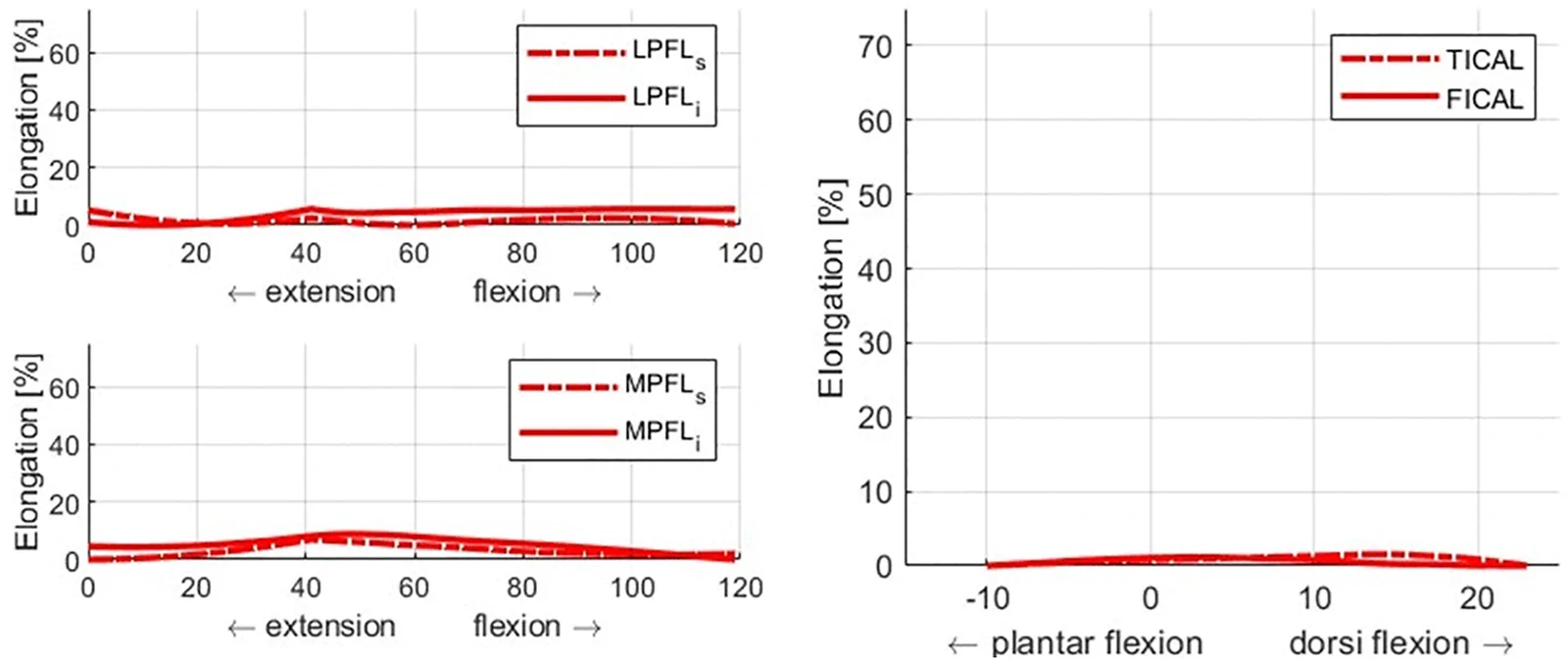
Percentage ligament elongation for the maximum congruence motion at the patella-femoral (left) and the ankle (right) joints.

Fig 9.a and 9.b report the percentual length variation for the patellofemoral and the ankle joints, respectively. Again, the maximum congruence motion is associated with ligament isometry, as reported in the literature for the considered ligaments [35,40].

Origin and insertions of all the ligaments, together with the computed resting length and isometry index, are reported in the supplementary material in xlsx format.

## Discussion

The goal of this paper was to update the TLEM 2.0 in terms of articular modeling. Using congruence maximization, the articular kinematics have been reconstructed from the shape of subchondral bone at the main articulations of the lower limb, namely the tibio-femoral, patella-femoral, talo-tibial, and calcaneo-talar joints. Compared with the provided revolute-based kinematics at the knee, the maximum congruence kinematics proved to be consistent with the anatomy of the donor: ligament behaviour was close to isometry for all the considered ligaments, with elongation well below 20% (Figs 7 and 8). On the contrary, revolute-based kinematics in combination with the original origins resulted in percentage elongation exceeding 60% for both the ACL and PCL, which raises serious concerns about the employability of this ligament selection. The same comparison was not possible for the other articulations, for which the original TLEM 2.0 provided no ligament data; however, ligament isometry provides an indirect validation of the quality of the predicted articular motion (Fig 9). Moreover, maximum congruence motion resulted in physiological contact patterns and homogeneous articular gaps, while the original revolute-based kinematics is associated with considerable disarticulation and bone-to-bone copenetration. Altogether, these results confirm the need for an improvement in the articular representation of the TLEM 2.0 and support the reliability of the maximum congruence kinematics as a valuable quantification of the donor’s actual motion used to develop the TLEM 2.0 dataset. It is important to note that no true joint kinematics data were originally recorded on the specimen. The image-guided surgery platform used to produce the dataset only allowed measurement of a single point position in 3D space relative to a fixed bone-mounted reference frame at any given time. Maximum congruence kinematics was therefore a valuable alternative to obtain the 3D joint kinematics of the TLEM 2.0 model.

This updated information opens several possible improvements of the MSK kinematic chain: 3D joint kinematics can be used directly, driving the secondary motion components as a function of the main articular DOF, as often done at the knee in MSK models such as [8,37]. The computed IHA can also be used to build moving-axis joint models [41–43]. These joint representations are particularly useful when the focus of MSK model is the analysis of articular response to load, where anatomical consistency is paramount for the correct evaluation of ligament and contact forces [4].

In association with articular kinematics, ligament insertions, contact points, and normals are also provided. Altogether, this information can be used to better quantify the contribution of the different articular structures to the joint response to load, for instance, by computing the ligament and contact forces capable of generating the joint reaction as computed by MSK model or by quantifying how compressive load is distributed between articular compartments [44]. Currently, this distribution is mostly computed in 2D and excludes ligament contributions [45].

Furthermore, the provided articular kinematics together with the associated isometric ligaments represent the basis for the definition of more advanced articular models, both rigid [25,46] or deformable [9,47–49], thus opening to more advanced MSK applications.

Alternatively, if a simpler kinematic chain would be suitable for the specific research question, the provided MHA could be employed. The ones proposed in this study have the merit of being computed from articular kinematics rather than derived from bone shape [50], potentially resulting in a better approximation of joint motion.

As for the neutral posture, it is worth noting that the same issues reported here were recognized by the researchers implementing the TLEM 2.0 model in a musculoskeletal software. The latter was based on the mean neutral ankle pose reported in the literature [51], while the knee was manually adjusted to compensate for the patellar tendon alignment. While these adjustments can improve neutral posture, they may still not be consistent with the donor’s specific articular anatomy. On the contrary, our approach derives the correction from the computed 3D joint kinematics: the final pose will therefore respect the physiological relations between articulating surfaces and the ligament behaviour.

As a final remark, it is worth noting that the presented approach, here applied to the donor geometry of the TLEM 2.0, is completely general and thus applicable to other musculoskeletal models or subjects. When the articular geometry of a subject is available, both from MRI or CT model, it is possible to reconstruct 3D joint kinematics as well as contact points and isometric ligaments following the same elaboration here reported, providing an alternative way for the personalization of the skeletal kinematic chain.

This work has limitations. The ligament at the knee shows better isometry than the revolute-based kinematics; however, the values are still higher than what was previously reported in the literature for the knee [33,34]. This may be due to a lack of representation of the menisci, normally included in previous implementations of the maximum congruence model of the knee [15]. This may amplify the uncertainty in the computed tibio-femoral kinematics. Although it could be improved, the overall geometry of the model, including the ligament one, is internally consistent and thus reliable, making it also possible to compute ligament forces from their directions. More detailed analyses of the ligament deformations under loads would instead require a deformable model and additional information on the ligament’s mechanical characteristics, which fall outside the scope of the study.

Joint kinematics have been determined by simulating cartilage as a constant layer over subchondral surfaces. This does not reflect the actual distribution of cartilage [38]. However, previous investigations proved that the impact of this assumption on the final kinematics is minor [22]. The same assumption may affect the estimated contact points and normals, as well as the chosen contact threshold value. Preliminary exploratory analyses in this study also confirmed low sensitivity, though a full analysis was not performed. Future analyses will assess the model’s sensitivity to these parameters.

The initial part of the patellar motion was obtained as the rigid act of motion connecting the measured CT scan patellar pose with the maximum congruence pose quantified at 40°. This choice may introduce some uncertainty and guarantees continuity only at the pose level, not at its derivative.

Finally, although the final musculoskeletal model is anatomically consistent and thus its outcomes are more reliable, it is built on a single donor. For this reason, its use as a generic model to be scaled on a wider population may fail to capture the specificity of some individuals. When possible, the personalization of the kinematic chain presented here should be applied to each patient.

Future work will address the implementation in the TLEM 2.0 and evaluation of updated joint models, such as knee and ankle parallel mechanisms synthesized from the provided 3D joint kinematics, articular contacts and ligaments, to be tested on available relevant datasets for the sake of comparison of the model performances with the rest of the literature [52].

## Supporting information

S1 File Supplementary material.rar. In this folder is included: the updated joint kinematics, computed through congruence maximization; the corresponding Mean Helical Axes of motion; the updated neutral posture; contact points and normals associated with the updated kinematics; origin and insertions of all the ligaments, together with the computed resting length and isometry index.
(RAR)
