# Supplementary material for: Update of the articular representation of the TLEM 2.0 musculoskeletal model
Source: PLoS One. 2026 Sep 21;21(9):e0358863. doi: 10.1371/journal.pone.0358863 (PMC13592602; doi:10.1371/journal.pone.0358863)
Supplement: Supplementary file 1 [file pone.0358863.s002.docx]

We thank the reviewer for their careful evaluation and constructive comment, which has helped us improve the manuscript.

Reviewer #1: The authors introduce a more realistic joint simulation and demonstrate that it produces superior qualitative results. However, as quantitative validation is still lacking, claims made in terms of 'more accurate estimation' seem somewhat exaggerated, and could be changed in 'more realistic'.

We agree with the reviewer on this point. We believe that a more appropriate statement is that the proposed model is more anatomically consistent and provides more reliable results. Although the underlying modelling approach has previously been validated against several experimental datasets, it has not been directly validated using the present dataset. We therefore revised the manuscript accordingly wherever appropriate.

In the following, some detailed comments are reported:- Abstract:- please remove claims of greater accuracy

Done

- please change 'physiologici' in 'more physiologic'

Done

- the authors should possibly report some numerical data (for example ligament strain for TLEM 2.0 vs improved TLEM)

Rather than reporting strain directly, we provide the percentage elongation of the most isometric ligament fiber for both the original TLEM model and the proposed updated model. The variation throughout the range of motion is reported in Figures 8 and 9. We chose not to report a single representative value, as maximum and minimum values may not accurately reflect the overall behavior of the models, whereas average values may mask important variations across the range of motion.

- Materials and methods

- this sentence sounds a bit obscure, the authors are kindly requested to make an effort to make it clearer "To obtain the initial part of the patello-femoral trajectory, the finite helical axis between the CT-scan pose from the database and the first one computed by congruence maximization was computed." How was it computed?

We apologize for the lack of clarity. We rephrased the sentence as “The initial part of the patellar trajectory was described as a rotation about and translation along the single finite helical axis that connects the patella pose relative to the femur measured from CT-scan to the first pose computed by congruence maximization at 40° of flexion: given the two rototranslational matrix, the finite axis of motion can be uniquely determined thanks to the Mozzi-Chasles’ theorem [31]”. We also added a reference to Tsai’s textbook. Among the many available resources describing the Mozzi–Chasles theorem, this reference provides a concise and clear explanation of how to compute the finite helical axis connecting two arbitrary poses of a rigid body. For the sake of readability, we chose not to include the detailed derivation in the manuscript.

- 'contact points and normals': the authors should possibly specify they are dealing with the normals on one of the two mating surfaces. A picture would be very helpful to clarify contact points and normals

Contact normals for each mating surface (femur and tibia) are computed using the estimated contact area derived from the proximity map. For each estimated contact area on each mating surface, the corresponding normal was computed as a weighted average of the corresponding facet normals. The weights were higher for most proximal points. The trajectories of the contact points are reported in Figure 7. We tried to add a similar figure for the normals, but we could not find a clear way to do so; thus, for the sake of readability, we chose not to display the contact normals. Nevertheless, their direction can be inferred from the local geometry of the mating surfaces.

- 'redefinition of ligaments': it is not clear how isometric condition was reached: is this a progressive optimization, starting from temptative positions of origin and insertions?

We apologize for the misunderstanding. When redefining ligament origins and insertions, we first identified a cloud of candidate points within each anatomical region. We then performed a numerical search to identify the pair of points, one within the origin region and one within the insertion region, that maximized ligament isometry. The manuscript has been revised accordingly to clarify this procedure.

- Discussion

- change 'advance' in 'advanced' in 'opening to more advanced MSK applications'

Done

- Figure 7 label

- typo, change 'trajectory con' in 'trajectory with'

Done

- Table 2

- measurement units for angles are missing

Added

Reviewer #2: The study titled “Update of the articular representation of the TLEM 2.0 musculoskeletal model” by Conconi and collegues, addresses a well-recognised gap in the TLEM 2.0 dataset. The methodology is sound and grounded in a well-validated framework, and the dataset produced is a meaningful contribution to the MSK modelling community. The manuscript falls within the scope of “Plos One” Journal.

We thank the reviewer for the nice comments and the appreciation of our work.

Before it can be considered for acceptance, authors are invited to address some points detailed below:

• In the Introduction, what do the authors refer to by "across different levels of MSK analysis"? The sentence appears ambiguous, and better precision on which “levels” the authors refer, would improve its clarity.

We agree with the reviewer that the original wording could have been clearer. We revised the text to explicitly identify the different stages of musculoskeletal analysis that may be affected by anatomical simplifications. The revised sentence now reads:“… across different levels of MSK analysis, from inverse kinematics, to inverse dynamics and static optimization, up to joint reaction computation”.

• The five bullet-point objectives listed in the Introduction are all well-addressed. However, the objective regarding mean helical axes (MHA) is almost entirely deferred to supplementary material without any result being shown or discussed in the main text. Either a brief summary result should be included, or the objective should be framed as an ancillary objective from the outset.

We thank the reviewer for this valuable comment, which helped us focus the manuscript more clearly. We agree that the computation of the mean helical axes represents a secondary aspect of the study rather than one of its main contributions. Accordingly, we revised the manuscript to reflect this perspective by merging the sections describing the definition of the neutral pose and the computation of the MHAs, and by reducing the emphasis on MHA analysis throughout the text.

• The assumption of simulating cartilage as a constant layer over subchondral surfaces may have a non-negligible impact on the downstream pipeline and results. Has a sensitivity analysis been performed regarding cartilage thickness? If not, could the authors comment on what variations would be expected, and to what extent such variations would affect the predicted kinematics, contact points, and ligament isometry?

We did not perform a formal sensitivity analysis on cartilage thickness, although we conducted exploratory tests by varying the subchondral bone offset around the selected values. The resulting changes were minimal. This observation is consistent with previous sensitivity analyses of the congruence-based model [1], which showed limited sensitivity of the predicted kinematics to offset variations. Moreover, we believe that the uncertainty we will necessarily introduce by assuming a constant layer of cartilage is justified by the generic nature of the model that we are proposing. In this context, the contribution of our work is to provide articular geometry, insertion locations, ligament resting length, and joint kinematics that are consistent with one another: motion is computed from articular geometry, while isometric ligaments are derived from the kinematics. The final model is thus internally consistent, while providing physiological motion and ligament properties. We cannot guarantee or quantify deviations from the actual behavior of the donor leg, but the model remains a valid representation of a generic leg.

On the other side, we could have applied a variable cartilage thickness. This, however, would have added uncertainty and variability to the model output, making it more difficult to identify a cartilage distribution that could be considered general. In this sense, it is worth noting that if we have a direct representation of the articular cartilage distribution, for instance, from MRI, we could apply the same approach, producing a more personalized kinematic chain of the leg.

We added some of these considerations to the discussion.

[1] Conconi, Michele, and Vincenzo Parenti Castelli. "Joint kinematics from functional adaptation: an application to the human ankle." *Applied Mechanics and Materials* 162 (2012): 266-275.

• The patello-femoral trajectory for flexion angles below 40° is obtained by interpolation via a finite helical axis rather than by congruence maximization. Why is 40° the threshold? What is the assumed continuity of the transition? Could this introduce artificial discontinuities in the kinematic trajectory at the junction point?

We thank the reviewer for the opportunity to clarify this point. During the initial phase of knee flexion, the patella is not fully engaged within the femoral trochlea and its motion is primarily governed by the forces transmitted through the quadriceps and patellar tendon. Under these conditions, the assumption of congruence maximization is not appropriate and therefore cannot be used to predict patellofemoral kinematics. Depending on the subject, full engagement of the patella within the trochlea generally occurs at approximately 30° of knee flexion. To ensure complete articular coupling, we therefore initiated the congruence-based computation at 40°.

The transition between the finite-helical-axis motion and the congruence-based motion is enforced to be continuous in both position and orientation, although not in their derivatives. Alternative interpolation schemes could impose higher-order continuity; however, in our opinion, the additional modeling assumptions they would require would introduce more uncertainty than benefit. Furthermore, patellar motion is known to be reasonably well approximated by a single axis of rotation during this portion of the movement. We have revised both the Methods and Discussion sections to clarify this rationale.

• A 2 mm distance threshold is used to define the contact area, but no rationale or sensitivity analysis is provided for this choice. Could the authors justify this value, or discuss whether a cartilage-thickness-relative threshold was considered? More broadly, could the authors indicate which steps in the pipeline are most sensitive to input assumptions, such as subchondral surface selection, ROM bounds, and cartilage thickness? According to the authors, which steps in the pipeline are most sensitive to input assumptions (subchondral surface selection, ROM bounds, cartilage thickness)?

We thank the reviewer for the careful analysis of our methodology. In the literature, several thresholds have been proposed to identify contact area by proximity, typically ranging from 0.5 to 2.5 mm. Some reviews can be found in ref [23] of the paper. In this sense, there is no general consensus on the optimal value. Again, we ran some tests on this specific parameter, which confirmed the results of [1]: while the threshold changes the amount of contact area that participates in the computation, the final contact points and normal soon become insensitive to its value. This makes sense, as our calculations weigh each area element's contribution based on its distance from the mating surface. We add a comment about it in the limitations section.

As for the most sensitive parameters, a thorough investigation would help address the point. Based on previous work done applying our predictive model to different articulations, we may say that in general the model is quite robust to the representation of the articular surfaces, the threshold chosen to define contact regions and congruence, initial pose at the beginning of the optimization, as well as the chosen independent variable to guide the optimization [1]. At the knee, however, the model typically requires a representation of the menisci to run properly, which are missing from the current implementation because no data for this anatomical structure were available in the TLEM2 dataset. It is therefore reasonable to infer that the current model could be sensitive to the subchondral selection and the distribution of the cartilage at the tibiofemoral joint. We added a comment when discussing the limitations of this work.

[1] Conconi, Michele, and Vincenzo Parenti Castelli. "Joint kinematics from functional adaptation: an application to the human ankle." *Applied Mechanics and Materials* 162 (2012): 266-275.

• The angular sampling step used for the independent DOF at each articulation is not reported in the manuscript. This information is relevant both for evaluating the resolution of the produced dataset and for ensuring reproducibility when the method is applied to other subjects, as suggested in the Discussion.

We apologize for this omission. The angular increment used in all computations was 1° for all joints. We have added this information to the Methods section.

• The comparison with the literature is predominantly qualitative ("in good agreement with previous literature"). Given that quantitative kinematic data are available in the supplementary material, it would be appropriate to provide at least a representative quantitative comparison, for example, mean and range of secondary motion components, with one or two key references for each joint. The current presentation makes it difficult for readers to assess agreement rigorously.

We agree with the reviewer that a quantitative comparison with a gold standard would be beneficial. The resulting kinematics for the various joints are reported quantitatively in the graphs of figures 2 and 4, which is the most common format adopted in the literature, and the numbers are indeed in agreement with previous literature.

We considered including an additional reference curve representing average literature data. However, we believe that such a comparison could be misleading. Kinematic descriptions depend strongly on the choice of reference frame, rotation parametrization, and subject-specific anatomy. Consequently, comparisons across datasets inevitably require transformations that may introduce bias and artificially improve or worsen the apparent agreement.

Moreover, the primary objective of this study was not to reproduce the exact kinematics of the donor specimen, but rather to generate joint motion that is anatomically consistent with the associated articular geometry. In this context, ligament elongation and contact point patterns provide an indirect but meaningful indicator of the physiological plausibility of the resulting kinematics.

• The comparison between revolute-based and maximum congruence kinematics is entirely visual (Figs. 2, 4). Why have the authors chosen to not address a quantitative approach?

We did not provide a single global metric, such as the root mean squared error, to summarize the difference between the two approaches. This choice was intentional. In the present study, we are not evaluating a model's error relative to a gold standard, since we do not have the true kinematics; rather, we are characterizing the differences between two distinct modeling approaches. A single metric would provide a more compact summary but would not add substantial information to the comparison. Furthermore, it could lead readers to interpret the reported value as a measure of model error.

The comparison shown in Figures 2 and 4 is nevertheless quantitative, since the differences between the two kinematic descriptions can be directly evaluated at each value of the independent degree of freedom by comparing the corresponding values on the y-axis.

• Figure 7 is described in the caption as showing contact point trajectories, but the corresponding Results section states only that results "are in agreement with what previously reported in the literature on active kinematics," without any further quantitative or qualitative analysis. The medial/lateral and anterior/posterior partitioning described in the Methods is not discussed in the Results. Could the authors provide at least a brief characterization of the contact point trajectories?

We thank the reviewer for giving us the opportunity to improve this section. We added a more detailed description of the contact point trajectories and compared them with observations previously reported in the literature.

• The entire dataset is derived from a single donor, which is an intrinsic limitation of the TLEM 2.0 framework and not a fault of the present work. However, the Discussion should address more explicitly how subject-specific variability in articular geometry might affect the predicted kinematics and the applicability of the dataset for general-purpose MSK modelling.

We fully agree with the reviewer on this point. The model is based on a single donor and is therefore representative of a specific individual. On the other hand, one could envisage building a musculoskeletal model based on average observations collected from a population of subjects, as was done, for example, for the Walker knee model. However, previous studies have shown that such an approach may produce anatomically inconsistent results, including cartilage interpenetration, excessive ligament elongation, and non-optimal muscle moment arms.

Based on these observations, our goal was to overcome these limitations by developing an internally consistent musculoskeletal model, thereby improving the reliability of its predictions. Furthermore, the methodology presented here is fully general and could be applied to derive a patient-specific musculoskeletal model whenever equivalent anatomical data become available for a new subject.

We added a dedicated discussion point to address this aspect.

• The Discussion makes a strong case for the superiority of congruence-based kinematics over revolute joints, which is well-supported. However, it does not engage with intermediate modelling approaches that are currently widespread in the MSK community, such as generalized joint coordinate models with coupled DOFs (e.g., the works by and Rajagopal et al. Guan et al., cited also by the authors themselves). If feasible, situating the proposed dataset in relation to these models would considerably improve the practical relevance of the Discussion.

Continuing the previous discussion, we agree that prescribed three-dimensional kinematics derived from population-average data may represent a valuable approach for modeling population-level behavior. However, this approach intrinsically limits analyses at the joint level because it cannot guarantee anatomical consistency. If average articular geometries, average ligament insertions, and average kinematics are combined, the resulting behavior may become non-physiological because these features do not scale linearly across subjects. As discussed in reference [3], this limitation may reduce the ability of such models to provide clinically meaningful information.

The possibility of using the updated information to drive the secondary motion components as a function of the main articular DOF and to build moving-axis joint models was already mentioned in the discussion. We consider this situates the proposed dataset in relation to these models. Although this is an interesting and important topic, we believe that a further detailed discussion would go beyond the scope of the present manuscript.

• The limitations are honestly stated, but the discussion of the absence of menisci is brief given its demonstrated impact on ligament isometry. The authors acknowledge that elongation values exceed literature benchmarks, but do not discuss whether the dataset is still usable for ligament-inclusive MSK models or whether corrective measures are needed. A more constructive framing would be valuable: for instance, what level of elongation is acceptable for specific modelling purposes, and under which conditions should the ligament data be used with caution?

We appreciate the reviewer’s observation. In this study, we would like to emphasize the elongation values of the original model relative to those of the new model. Although our values remain suboptimal in some cases, we were nevertheless able to improve ligament behavior, thus model consistency, despite the absence of a direct representation of the donor’s soft tissues in the TLEM dataset.

Our model treats the knee, as well as the other joints, as a rigid joint. The predicted elongation corresponds to the joint’s natural path independently of any external or muscle loads. Consequently, ligament elongation should be interpreted primarily as an indicator of anatomical consistency rather than as a direct quantification of joint behavior during tasks such as walking, jumping, and other dynamic activities. In this sense, the motion still shows slight inconsistency with soft tissues, though it has improved significantly compared with the previous model: the geometric information of the model, including ligament ones, can be considered reliable, for instance, to also obtain the direction of ligament forces in the joint and their average length. If one were interested in evaluating ligament mechanics in detail, including changes in ligament length under load, a deformable model would be needed. This, however, would require additional ligament information, such as stiffness and unloaded lengths, to position the actual ligament elongation relative to its tension. In this sense, since we do not have this information, the effect of the measured ligament elongations at some flexion angle is less predictable if that is used to compute ligament forces from the ligament mechanical characteristics.

We have added a brief clarification in the discussion section.

• In Figure 8-9, the direction of the flexion/extension arrows on the x-axis lacks clarity. While for the knee joint the axis origin (0°) is expected to correspond to full extension, the equivalent reference configuration for the ankle is not immediately apparent. Could the authors clarify the angular convention adopted for each joint?

We apologize for the lack of clarity. In the figures, the arrows are intended to indicate the direction of motion along the horizontal axis. Moving from left to right (i.e., increasing the x-coordinate) corresponds to increasing knee flexion and ankle dorsiflexion, respectively.

Regarding the reference configuration, we adopted the coordinate system and conventions defined in the TLEM model. For both the knee and the ankle, a flexion angle of 0° corresponds to the neutral position, namely full knee extension and a foot posture with the plantar surface perpendicular to the longitudinal axis of the tibia.

• There is a typesetting error in the Methods section: the reference appearing as "[conconisancisi-2024]" should likely correspond to reference [22]. Please verify and correct accordingly. Similarly, the reference appearing as "[woltring-1990]" should likely correspond to reference [53]. Please verify and correct accordingly. There is a typographical error in the caption of Figure 7, which reads "trajectory con contact points." The word "con" should be removed.

We thank the reviewer for identifying these typographical errors. They have now been corrected.
