# Supplementary material for: Update of the articular representation of the TLEM 2.0 musculoskeletal model
Source: PLoS One. 2026 Sep 21;21(9):e0358863. doi: 10.1371/journal.pone.0358863 (PMC13592602; doi:10.1371/journal.pone.0358863)
Supplement: Peer review history

# Peer review: Decision Letter 0

**DOI:** 10.1371/journal.pone.0358863.r001

**Authors:** Giulia Pascoletti

27 May 2026

Dear Dr. Conconi,

Thank you for submitting your manuscript to PLOS ONE. After careful consideration, we feel that it has merit but does not fully meet PLOS ONE’s publication criteria as it currently stands. Therefore, we invite you to submit a revised version of the manuscript that addresses the points raised during the review process.

The reviewers have pointed out that the manuscript would benefit from clearer methodological descriptions and deeper discussion of modelling assumptions and limitations.

Please submit your revised manuscript by Jul 11 2026 11:59PM. If you will need more time than this to complete your revisions, please reply to this message or contact the journal office at plosone@plos.org. When you're ready to submit your revision, log on to https://www.editorialmanager.com/pone/ and select the 'Submissions Needing Revision' folder to locate your manuscript file.

- A letter that responds to each point raised by the academic editor and reviewer(s). You should upload this letter as a separate file labeled 'Response to Reviewers'.
- A marked-up copy of your manuscript that highlights changes made to the original version. You should upload this as a separate file labeled 'Revised Manuscript with Track Changes'.
- An unmarked version of your revised paper without tracked changes. You should upload this as a separate file labeled 'Manuscript'.

If you would like to make changes to your financial disclosure, please include your updated statement in your cover letter. Guidelines for resubmitting your figure files are available below the reviewer comments at the end of this letter.

If applicable, we recommend that you deposit your laboratory protocols in protocols.io to enhance the reproducibility of your results. Protocols.io assigns your protocol its own identifier (DOI) so that it can be cited independently in the future. For instructions see: https://journals.plos.org/plosone/s/submission-guidelines#loc-laboratory-protocols. Additionally, PLOS ONE offers an option for publishing peer-reviewed Lab Protocol articles, which describe protocols hosted on protocols.io. Read more information on sharing protocols at https://plos.org/protocols?utm_medium=editorial-email&utm_source=authorletters&utm_campaign=protocols.

As the corresponding author, your ORCID iD is verified in the submission system and will appear in the published article. PLOS supports the use of ORCID, and we encourage all coauthors to register for an ORCID iD and use it as well. Please encourage your coauthors to verify their ORCID iD within the submission system before final acceptance, as unverified ORCID iDs will not appear in the published article. *Only*  the individual author can complete the verification step; PLOS staff *cannot*  verify ORCID iDs on behalf of authors.

We look forward to receiving your revised manuscript.

Kind regards,

Giulia Pascoletti, Ph.D.

Academic Editor

PLOS One

**Journal Requirements:**

When submitting your revision, we need you to address these additional requirements.

1. Please ensure that your manuscript meets PLOS ONE's style requirements, including those for file naming. The PLOS ONE style templates can be found at

https://journals.plos.org/plosone/s/file?id=wjVg/PLOSOne_formatting_sample_main_body.pdf and

https://journals.plos.org/plosone/s/file?id=ba62/PLOSOne_formatting_sample_title_authors_affiliations.pdf

2. Please include your tables as part of your main manuscript and remove the individual files. Please note that supplementary tables (should remain/ be uploaded) as separate "supporting infiormation" files.

3. Thank you for stating in your Funding Statement:

“This work was partially supported by the “PHC GALILEE” programme (project number: 48932SG), funded by the French Ministry for Europe and Foreign Affairs, the French Ministry for Higher Education and Research and the Italian Ministero dell’Università e della Ricerca (MUR).”

Please provide an amended statement that declares *all* the funding or sources of support (whether external or internal to your organization) received during this study, as detailed online in our guide for authors at http://journals.plos.org/plosone/s/submit-now.  Please also include the statement “There was no additional external funding received for this study.” in your updated Funding Statement.

Please include your amended Funding Statement within your cover letter. We will change the online submission form on your behalf.

4. Please state what role the funders took in the study.  If the funders had no role, please state: "The funders had no role in study design, data collection and analysis, decision to publish, or preparation of the manuscript."

If this statement is not correct you must amend it as needed.

Please include this amended Role of Funder statement in your cover letter; we will change the online submission form on your behalf.

5. Please note that funding information should not appear in any section or other areas of your manuscript. We will only publish funding information present in the Funding Statement section of the online submission form. Please remove any funding-related text from the manuscript.

6. Please note that your Data Availability Statement is currently missing the repository name. If your manuscript is accepted for publication, you will be asked to provide these details on a very short timeline. We therefore suggest that you provide this information now, though we will not hold up the peer review process if you are unable.

7. Please upload a new copy of Figures 2, 4, 8 and 9 as the detail is not clear. Please follow the link for more information:  https://journals.plos.org/plosone/s/figures

8. Please include captions for your Supporting Information files at the end of your manuscript, and update any in-text citations to match accordingly. Please see our Supporting Information guidelines for more information: http://journals.plos.org/plosone/s/supporting-information.

9. If the reviewer comments include a recommendation to cite specific previously published works, please review and evaluate these publications to determine whether they are relevant and should be cited. There is no requirement to cite these works unless the editor has indicated otherwise.

[Note: HTML markup is below. Please do not edit.]

Reviewers' comments:

Reviewer's Responses to Questions

**Comments to the Author**

1. Is the manuscript technically sound, and do the data support the conclusions?

Reviewer #1: Partly

Reviewer #2: Yes

**********

2. Has the statistical analysis been performed appropriately and rigorously?

Reviewer #1: N/A

Reviewer #2: No

**********

3. Have the authors made all data underlying the findings in their manuscript fully available?

The PLOS Data policy

Reviewer #1: Yes

Reviewer #2: Yes

**********

4. Is the manuscript presented in an intelligible fashion and written in standard English?

Reviewer #1: Yes

Reviewer #2: Yes

**********

Reviewer #1: The authors introduce a more realistic joint simulation and demonstrate that it produces superior qualitative results.

However, as quantitative validation is still lacking, claims made in terms of 'more accurate estimation' seem somewhat exaggerated, and could be changed in 'more realistic'. In the following, some detailed comments are reported:

- Abstract:

- please remove claims of greater accuracy

- please change 'physiologici' in 'more physiologic'

- the authors should possibly report some numerical data (for example ligament strain for TLEM 2.0 vs improved TLEM)

- Materials and methods

- this sentence sounds a bit obscure, the authors are kindly requested to make an effort to make it clearer "To obtain the

initial part of the patello-femoral trajectory, the finite helical axis between the CT-scan pose from the database and the

first one computed by congruence maximization was computed." How was it computed?

- 'contact points and normals': the authors should possibly specify they are dealing with the normals on one of the two

mating surfaces. A picture would be very helpful to clarify contact points and normals

- 'ridefinition of ligaments': it is not clear how isometric condition was reached: is this a progressive optiization, starting

from temptative positions of origin and insertions?

- Discussion

- change 'advance' in 'advanced' in 'opening to more advanced MSK applications'

- Figure 7 label

- typo, change 'trajectory con' in 'trajectory with'

- Table 2

- measurement units for angles are missing

Reviewer #2: The study titled “Update of the articular representation of the TLEM 2.0 musculoskeletal model” by Conconi and collegues, addresses a well-recognised gap in the TLEM 2.0 dataset. The methodology is sound and grounded in a well-validated framework, and the dataset produced is a meaningful contribution to the MSK modelling community. The manuscript falls within the scope of “Plos One” Journal. Before it can be considered for acceptance, authors are invited to address some points detailed below:

• In the Introduction, what do the authors refer to by "across different levels of MSK analysis"? The sentence appears ambiguous, and better precision on which “levels” the authors refer, would improve its clarity.

• The five bullet-point objectives listed in the Introduction are all well-addressed. However, the objective regarding mean helical axes (MHA) is almost entirely deferred to supplementary material without any result being shown or discussed in the main text. Either a brief summary result should be included, or the objective should be framed as an ancillary objective from the outset.

• The assumption of simulating cartilage as a constant layer over subchondral surfaces may have a non-negligible impact on the downstream pipeline and results. Has a sensitivity analysis been performed regarding cartilage thickness? If not, could the authors comment on what variations would be expected, and to what extent such variations would affect the predicted kinematics, contact points, and ligament isometry?

• The patello-femoral trajectory for flexion angles below 40° is obtained by interpolation via a finite helical axis rather than by congruence maximization. Why is 40° the threshold? What is the assumed continuity of the transition? Could this introduce artificial discontinuities in the kinematic trajectory at the junction point?

• A 2 mm distance threshold is used to define the contact area, but no rationale or sensitivity analysis is provided for this choice. Could the authors justify this value, or discuss whether a cartilage-thickness-relative threshold was considered?

More broadly, could the authors indicate which steps in the pipeline are most sensitive to input assumptions, such as subchondral surface selection, ROM bounds, and cartilage thickness?

• The angular sampling step used for the independent DOF at each articulation is not reported in the manuscript. This information is relevant both for evaluating the resolution of the produced dataset and for ensuring reproducibility when the method is applied to other subjects, as suggested in the Discussion.

• The comparison with the literature is predominantly qualitative ("in good agreement with previous literature"). Given that quantitative kinematic data are available in the supplementary material, it would be appropriate to provide at least a representative quantitative comparison, for example, mean and range of secondary motion components, with one or two key references for each joint. The current presentation makes it difficult for readers to assess agreement rigorously.

• The comparison between revolute-based and maximum congruence kinematics is entirely visual (Figs. 2, 4). Why have the authors chosen to not address a quantitative approach?

• Similar to some point before, a 2 mm distance threshold for defining the contact area is used. Could the authors please provide the rationale or sensitivity analysis that are behind this choice? According to the authors, which steps in the pipeline are most sensitive to input assumptions (subchondral surface selection, ROM bounds, cartilage thickness)?

• Figure 7 is described in the caption as showing contact point trajectories, but the corresponding Results section states only that results "are in agreement with what previously reported in the literature on active kinematics," without any further quantitative or qualitative analysis. The medial/lateral and anterior/posterior partitioning described in the Methods is not discussed in the Results. Could the authors provide at least a brief characterization of the contact point trajectories?

• The entire dataset is derived from a single donor, which is an intrinsic limitation of the TLEM 2.0 framework and not a fault of the present work. However, the Discussion should address more explicitly how subject-specific variability in articular geometry might affect the predicted kinematics and the applicability of the dataset for general-purpose MSK modelling.

• The Discussion makes a strong case for the superiority of congruence-based kinematics over revolute joints, which is well-supported. However, it does not engage with intermediate modelling approaches that are currently widespread in the MSK community, such as generalized joint coordinate models with coupled DOFs (e.g., the works by and Rajagopal et al. Guan et al., cited also by the authors themselves). If feasible, situating the proposed dataset in relation to these models would considerably improve the practical relevance of the Discussion.

• The limitations are honestly stated, but the discussion of the absence of menisci is brief given its demonstrated impact on ligament isometry. The authors acknowledge that elongation values exceed literature benchmarks, but do not discuss whether the dataset is still usable for ligament-inclusive MSK models or whether corrective measures are needed. A more constructive framing would be valuable: for instance, what level of elongation is acceptable for specific modelling purposes, and under which conditions should the ligament data be used with caution?

• In Figure 8-9, the direction of the flexion/extension arrows on the x-axis lacks clarity. While for the knee joint the axis origin (0°) is expected to correspond to full extension, the equivalent reference configuration for the ankle is not immediately apparent. Could the authors clarify the angular convention adopted for each joint?

• There is a typesetting error in the Methods section: the reference appearing as "[conconisancisi-2024]" should likely correspond to reference [22]. Please verify and correct accordingly.

Similarly, the reference appearing as "[woltring-1990]" should likely correspond to reference [53]. Please verify and correct accordingly.

There is a typographical error in the caption of Figure 7, which reads "trajectory con contact points." The word "con" should be removed.

**********

what does this mean?). If published, this will include your full peer review and any attached files.

If you choose “no”, your identity will remain anonymous but your review may still be made public.

**Do you want your identity to be public for this peer review?**  For information about this choice, including consent withdrawal, please see our Privacy Policy

Reviewer #1: No

Reviewer #2: No

**********

[NOTE: If reviewer comments were submitted as an attachment file, they will be attached to this email and accessible via the submission site. Please log into your account, locate the manuscript record, and check for the action link "View Attachments". If this link does not appear, there are no attachment files.]

To ensure your figures meet our technical requirements, please review our figure guidelines: https://journals.plos.org/plosone/s/figures

You may also use PLOS’s free figure tool, NAAS, to help you prepare publication quality figures: https://journals.plos.org/plosone/s/figures#loc-tools-for-figure-preparation.

NAAS will assess whether your figures meet our technical requirements by comparing each figure against our figure specifications.

# Author comment: Author response to Decision Letter 1

**DOI:** 10.1371/journal.pone.0358863.r002

21 Aug 2026

We thank the reviewer for their careful evaluation and constructive comment, which has helped us improve the manuscript.

Reviewer #1: The authors introduce a more realistic joint simulation and demonstrate that it produces superior qualitative results. However, as quantitative validation is still lacking, claims made in terms of 'more accurate estimation' seem somewhat exaggerated, and could be changed in 'more realistic'.

We agree with the reviewer on this point. We believe that a more appropriate statement is that the proposed model is more anatomically consistent and provides more reliable results. Although the underlying modelling approach has previously been validated against several experimental datasets, it has not been directly validated using the present dataset. We therefore revised the manuscript accordingly wherever appropriate.

In the following, some detailed comments are reported:- Abstract:- please remove claims of greater accuracy

Done

- please change 'physiologici' in 'more physiologic'

Done

- the authors should possibly report some numerical data (for example ligament strain for TLEM 2.0 vs improved TLEM)

Rather than reporting strain directly, we provide the percentage elongation of the most isometric ligament fiber for both the original TLEM model and the proposed updated model. The variation throughout the range of motion is reported in Figures 8 and 9. We chose not to report a single representative value, as maximum and minimum values may not accurately reflect the overall behavior of the models, whereas average values may mask important variations across the range of motion.

- Materials and methods

- this sentence sounds a bit obscure, the authors are kindly requested to make an effort to make it clearer "To obtain the initial part of the patello-femoral trajectory, the finite helical axis between the CT-scan pose from the database and the first one computed by congruence maximization was computed." How was it computed?

We apologize for the lack of clarity. We rephrased the sentence as “The initial part of the patellar trajectory was described as a rotation about and translation along the single finite helical axis that connects the patella pose relative to the femur measured from CT-scan to the first pose computed by congruence maximization at 40° of flexion: given the two rototranslational matrix, the finite axis of motion can be uniquely determined thanks to the Mozzi-Chasles’ theorem [31]”. We also added a reference to Tsai’s textbook. Among the many available resources describing the Mozzi–Chasles theorem, this reference provides a concise and clear explanation of how to compute the finite helical axis connecting two arbitrary poses of a rigid body. For the sake of readability, we chose not to include the detailed derivation in the manuscript.

- 'contact points and normals': the authors should possibly specify they are dealing with the normals on one of the two mating surfaces. A picture would be very helpful to clarify contact points and normals

Contact normals for each mating surface (femur and tibia) are computed using the estimated contact area derived from the proximity map. For each estimated contact area on each mating surface, the corresponding normal was computed as a weighted average of the corresponding facet normals. The weights were higher for most proximal points. The trajectories of the contact points are reported in Figure 7. We tried to add a similar figure for the normals, but we could not find a clear way to do so; thus, for the sake of readability, we chose not to display the contact normals. Nevertheless, their direction can be inferred from the local geometry of the mating surfaces.

- 'redefinition of ligaments': it is not clear how isometric condition was reached: is this a progressive optimization, starting from temptative positions of origin and insertions?

We apologize for the misunderstanding. When redefining ligament origins and insertions, we first identified a cloud of candidate points within each anatomical region. We then performed a numerical search to identify the pair of points, one within the origin region and one within the insertion region, that maximized ligament isometry. The manuscript has been revised accordingly to clarify this procedure.

- Discussion

- change 'advance' in 'advanced' in 'opening to more advanced MSK applications'

Done

- Figure 7 label

- typo, change 'trajectory con' in 'trajectory with'

Done

- Table 2

- measurement units for angles are missing

Added Reviewer #2: The study titled “Update of the articular representation of the TLEM 2.0 musculoskeletal model” by Conconi and collegues, addresses a well-recognised gap in the TLEM 2.0 dataset. The methodology is sound and grounded in a well-validated framework, and the dataset produced is a meaningful contribution to the MSK modelling community. The manuscript falls within the scope of “Plos One” Journal.

We thank the reviewer for the nice comments and the appreciation of our work.

Before it can be considered for acceptance, authors are invited to address some points detailed below:

• In the Introduction, what do the authors refer to by "across different levels of MSK analysis"? The sentence appears ambiguous, and better precision on which “levels” the authors refer, would improve its clarity.

We agree with the reviewer that the original wording could have been clearer. We revised the text to explicitly identify the different stages of musculoskeletal analysis that may be affected by anatomical simplifications. The revised sentence now reads:“… across different levels of MSK analysis, from inverse kinematics, to inverse dynamics and static optimization, up to joint reaction computation”.

• The five bullet-point objectives listed in the Introduction are all well-addressed. However, the objective regarding mean helical axes (MHA) is almost entirely deferred to supplementary material without any result being shown or discussed in the main text. Either a brief summary result should be included, or the objective should be framed as an ancillary objective from the outset.

We thank the reviewer for this valuable comment, which helped us focus the manuscript more clearly. We agree that the computation of the mean helical axes represents a secondary aspect of the study rather than one of its main contributions. Accordingly, we revised the manuscript to reflect this perspective by merging the sections describing the definition of the neutral pose and the computation of the MHAs, and by reducing the emphasis on MHA analysis throughout the text.

• The assumption of simulating cartilage as a constant layer over subchondral surfaces may have a non-negligible impact on the downstream pipeline and results. Has a sensitivity analysis been performed regarding cartilage thickness? If not, could the authors comment on what variations would be expected, and to what extent such variations would affect the predicted kinematics, contact points, and ligament isometry?

We did not perform a formal sensitivity analysis on cartilage thickness, although we conducted exploratory tests by varying the subchondral bone offset around the selected values. The resulting changes were minimal. This observation is consistent with previous sensitivity analyses of the congruence-based model [1], which showed limited sensitivity of the predicted kinematics to offset variations. Moreover, we believe that the uncertainty we will necessarily introduce by assuming a constant layer of cartilage is justified by the generic nature of the model that we are proposing. In this context, the contribution of our work is to provide articular geometry, insertion locations, ligament resting length, and joint kinematics that are consistent with one another: motion is computed from articular geometry, while isometric ligaments are derived from the kinematics. The final model is thus internally consistent, while providing physiological motion and ligament properties. We cannot guarantee or quantify deviations from the actual behavior of the donor leg, but the model remains a valid representation of a generic leg.

On the other side, we could have applied a variable cartilage thickness. This, however, would have added uncertainty and variability to the model output, making it more difficult to identify a cartilage distribution that could be considered general. In this sense, it is worth noting that if we have a direct representation of the articular cartilage distribution, for instance, from MRI, we could apply the same approach, producing a more personalized kinematic chain of the leg.

We added some of these considerations to the discussion.

[1] Conconi, Michele, and Vincenzo Parenti Castelli. "Joint kinematics from functional adaptation: an application to the human ankle." Applied Mechanics and Materials 162 (2012): 266-275.

• The patello-femoral trajectory for flexion angles below 40° is obtained by interpolation via a finite helical axis rather than by congruence maximization. Why is 40° the threshold? What is the assumed continuity of the transition? Could this introduce artificial discontinuities in the kinematic trajectory at the junction point?

We thank the reviewer for the opportunity to clarify this point. During the initial phase of knee flexion, the patella is not fully engaged within the femoral trochlea and its motion is primarily governed by the forces transmitted through the quadriceps and patellar tendon. Under these conditions, the assumption of congruence maximization is not appropriate and therefore cannot be used to predict patellofemoral kinematics. Depending on the subject, full engagement of the patella within the trochlea generally occurs at approximately 30° of knee flexion. To ensure complete articular coupling, we therefore initiated the congruence-based computation at 40°.

The transition between the finite-helical-axis motion and the congruence-based motion is enforced to be continuous in both position and orientation, although not in their derivatives. Alternative interpolation schemes could impose higher-order continuity; however, in our opinion, the additional modeling assumptions they would require would introduce more uncertainty than benefit. Furthermore, patellar motion is known to be reasonably well approximated by a single axis of rotation during this portion of the movement. We have revised both the Methods and Discussion sections to clarify this rationale.

• A 2 mm distance threshold is used to define the contact area, but no rationale or sensitivity analysis is provided for this choice. Could the authors justify this value, or discuss whether a cartilage-thickness-relative threshold was considered? More broadly, could the authors indicate which steps in the pipeline are most sensitive to input assumptions, such as subchondral surface selection, ROM bounds, and cartilage thickness? According to the authors, which steps in the pipeline are most sensitive to input assumptions (subchondral surface selection, ROM bounds, cartilage thickness)?

We thank the reviewer for the careful analysis of our methodology. In the literature, several thresholds have been proposed to identify contact area by proximity, typically ranging from 0.5 to 2.5 mm. Some reviews can be found in ref [23] of the paper. In this sense, there is no general consensus on the optimal value. Again, we ran some tests on this specific parameter, which confirmed the results of [1]: while the threshold changes the amount of contact area that participates in the computation, the final contact points and normal soon become insensitive to its value. This makes sense, as our calculations weigh each area element's contribution based on its distance from the mating surface. We add a comment about it in the limitations section.

As for the most sensitive parameters, a thorough investigation would help address the point. Based on previous work done applying our predictive model to different articulations, we may say that in general the model is quite robust to the representation of the articular surfaces, the threshold chosen to define contact regions and congruence, initial pose at the beginning of the optimization, as well as the chosen independent variable to guide the optimization [1]. At the knee, however, the model typically requires a representation of the menisci to run properly, which are missing from the current implementation because no data for this anatomical structure were available in the TLEM2 dataset. It is therefore reasonable to infer that the current model could be sensitive to the subchondral selection and the distribution of the cartilage at the tibiofemoral joint. We added a comment when discussing the limitations of this work.

[1] Conconi, Michele, and Vincenzo Parenti Castelli. "Joint kinematics from functional adaptation: an application to the human ankle." Applied Mechanics and Materials 162 (2012): 266-275.

• The angular sampling step used for the independent DOF at each articulation is not reported in the manuscript. This information is relevant both for evaluating the resolution of the produced dataset and for ensuring reproducibility when the method is applied to other subjects, as suggested in the Discussion.

We apologize for this omission. The angular increment used in all computations was 1° for all joints. We have added this information to the Methods section.

• The comparison with the literature is predominantly qualitative ("in good agreement with previous literature"). Given that quantitative kinematic data are available in the supplementary material, it would be appropriate to provide at least a representative quantitative comparison, for example, mean and range of secondary motion components, with one or two key references for each joint. The current presentation makes it difficult for readers to assess agreement rigorously.

We agree with the reviewer that a quantitative comparison with a gold standard would be beneficial. The resulting kinematics for the various joints are reported quantitatively in the graphs of figures 2 and 4, which is the most common format adopted in the literature, and the numbers are indeed in agreement with previous literature.

We considered including an additional reference curve representing average literature data. However, we believe that such a comparison could be misleading. Kinematic descriptions depend strongly on the choice of reference frame, rotation parametrization, and subject-specific anatomy. Consequently, comparisons across datasets inevitably require transformations that may introduce bias and artificially improve or worsen the apparent agreement.

Moreover, the primary objective of this study was not to reproduce the exact kinematics of the donor specimen, but rather to generate joint motion that is anatomically consistent with the associated articular geometry. In this context, ligament elongation and contact point patterns provide an indirect but meaningful indicator of the physiological plausibility of the resulting kinematics.

• The comparison between revolute-based and maximum congruence kinematics is entirely visual (Figs. 2, 4). Why have the authors chosen to not address a quantitative approach?

We did not provide a single global metric, such as the root mean squared error, to summarize the difference between the two approaches. This choice was intentional. In the present study, we are not evaluating a model's error relative to a gold standard, since we do not have the true kinematics; rather, we are characterizing the differences between two distinct modeling approaches. A single metric would provide a more compact summary but would not add substantial information to the comparison. Furthermore, it could lead readers to interpret the reported value as a measure of model error.

The comparison shown in Figures 2 and 4 is nevertheless quantitative, since the differences between the two kinematic descriptions can be directly evaluated at each value of the independent degree of freedom by comparing the corresponding values on the y-axis.

• Figure 7 is described in the caption as showing contact point trajectories, but the corresponding Results section states only that results "are in agreement with what previously reported in the literature on active kinematics," without any further quantitative or qualitative analysis. The medial/lateral and anterior/posterior partitioning described in the Methods is not discussed in the Results. Could the authors provide at least a brief characterization of the contact point trajectories?

We thank the reviewer for giving us the opportunity to improve this section. We added a more detailed description of the contact point trajectories and compared them with observations previously reported in the literature.

• The entire dataset is derived from a single donor, which is an intrinsic limitation of the TLEM 2.0 framework and not a fault of the present work. However, the Discussion should address more explicitly how subject-specific variability in articular geometry might affect the predicted kinematics and the applicability of the dataset for general-purpose MSK modelling.

We fully agree with the reviewer on this point. The model is based on a single donor and is therefore representative of a specific individual. On the other hand, one could envisage building a musculoskeletal model based on average observations collected from a population of subjects, as was done, for example, for the Walker knee model. However, previous studies have shown that such an approach may produce anatomically inconsistent results, including cartilage interpenetration, excessive ligament elongation, and non-optimal muscle moment arms.

Based on these observations, our goal was to overcome these limitations by developing an internally consistent musculoskeletal model, thereby improving the reliability of its predictions. Furthermore, the methodology presented here is fully general and could be applied to derive a patient-specific musculoskeletal model whenever equivalent anatomical data become available for a new subject.

We added a dedicated discussion point to address this aspect.

• The Discussion makes a strong case for the superiority of congruence-based kinematics over revolute joints, which is well-supported. However, it does not engage with intermediate modelling approaches that are currently widespread in the MSK community, such as generalized joint coordinate models with coupled DOFs (e.g., the works by and Rajagopal et al. Guan et al., cited also by the authors themselves). If feasible, situating the proposed dataset in relation to these models would considerably improve the practical relevance of the Discussion.

Continuing the previous discussion, we agree that prescribed three-dimensional kinematics derived from population-average data may represent a valuable approach for modeling population-level behavior. However, this approach intrinsically limits analyses at the joint level because it cannot guarantee anatomical consistency. If average articular geometries, average ligament insertions, and average kinematics are combined, the resulting behavior may become non-physiological because these features do not scale linearly across subjects. As discussed in reference [3], this limitation may reduce the ability of such models to provide clinically meaningful information.

The possibility of using the updated information to drive the secondary motion components as a function of the main articular DOF and to build moving-axis joint models was already mentioned in the discussion. We consider this situates the proposed dataset in relation to these models. Although this is an interesting and important topic, we believe that a further detailed discussion would go beyond the scope of the present manuscript.

• The limitations are honestly stated, but the discussion of the absence of menisci is brief given its demonstrated impact on ligament isometry. The authors acknowledge that elongation values exceed literature benchmarks, but do not discuss whether the dataset is still usable for ligament-inclusive MSK models or whether corrective measures are needed. A more constructive framing would be valuable: for instance, what level of elongation is acceptable for specific modelling purposes, and under which conditions should the ligament data be used with caution?

We appreciate the reviewer’s observation. In this study, we would like to emphasize the elongation values of the original model relative to those of the new model. Although our values remain suboptimal in some cases, we were nevertheless able to improve ligament behavior, thus model consistency, despite the absence of a direct representation of the donor’s soft tissues in the TLEM dataset.

Our model treats the knee, as well as the other joints, as a rigid joint. The predicted elongation corresponds to the joint’s natural path independently of any external or muscle loads. Consequently, ligament elongation should be interpreted primarily as an indicator of anatomical consistency rather than as a direct quantification of joint behavior during tasks such as walking, jumping, and other dynamic activities. In this sense, the motion still shows slight inconsistency with soft tissues, though it has improved significantly compared with the previous model: the geometric information of the model, including ligament ones, can be considered reliable, for instance, to also obtain the direction of ligament forces in the joint and their average length. If one were interested in evaluating ligament mechanics in detail, including changes in ligament length under load, a deformable model would be needed. This, however, would require additional ligament information, such as stiffness and unloaded lengths, to position the actual ligament elongation relative to its tension. In this sense, since we do not have this information, the effect of the measured ligament elongations at some flexion angle is less predictable if that is used to compute ligament forces from the ligament mechanical characteristics.

We have added a brief clarification in the discussion section.

• In Figure 8-9, the direction of the flexion/extension arrows on the x-axis lacks clarity. While for the knee joint the axis origin (0°) is expected to correspond to full extension, the equivalent reference configuration for the ankle is not immediately apparent. Could the authors clarify the angular convention adopted for each joint?

We apologize for the lack of clarity. In the figures, the arrows are intended to indicate the direction of motion along the horizontal axis. Moving from left to right (i.e., increasing the x-coordinate) corresponds to increasing knee flexion and ankle dorsiflexion, respectively.

Regarding the reference configuration, we adopted the coordinate system and conventions defined in the TLEM model. For both the knee and the ankle, a flexion angle of 0° corresponds to the neutral position, namely full knee extension and a foot posture with the plantar surface perpendicular to the longitudinal axis of the tibia.

• There is a typesetting error in the Methods section: the reference appearing as "[conconisancisi-2024]" should likely correspond to reference [22]. Please verify and correct accordingly. Similarly, the reference appearing as "[woltring-1990]" should likely correspond to reference [53]. Please verify and correct accordingly. There is a typographical error in the caption of Figure 7, which reads "trajectory con contact points." The word "con" should be removed.

We thank the reviewer for identifying these typographical errors. They have now been corrected.

Attachment Submitted filename: Reply to Reviewers.docx

# Peer review: Decision Letter 1

**DOI:** 10.1371/journal.pone.0358863.r003

**Authors:** Rohan Kothurkar

7 Sep 2026

Update of the articular representation of the TLEM 2.0 musculoskeletal model

PONE-D-26-04622R1

Dear Dr. Conconi,

We’re pleased to inform you that your manuscript has been judged scientifically suitable for publication and will be formally accepted for publication once it meets all outstanding technical requirements.

Within one week, you’ll receive an e-mail detailing the required amendments. When these have been addressed, you’ll receive a formal acceptance letter and your manuscript will be scheduled for publication.

An invoice will be generated when your article is formally accepted. Please note, if your institution has a publishing partnership with PLOS and your article meets the relevant criteria, all or part of your publication costs will be covered. Please make sure your user information is up-to-date by logging into Editorial Manager at Editorial Manager® and clicking the ‘Update My Information' link at the top of the page. For questions related to billing, please contact billing support.

If your institution or institutions have a press office, please notify them about your upcoming paper to help maximize its impact. If they’ll be preparing press materials, please inform our press team as soon as possible -- no later than 48 hours after receiving the formal acceptance. Your manuscript will remain under strict press embargo until 2 pm Eastern Time on the date of publication. For more information, please contact onepress@plos.org.

Kind regards,

Rohan Kothurkar, PhD

Academic Editor

PLOS One

Additional Editor Comments (optional):

Reviewers' comments:

Reviewer's Responses to Questions

**Comments to the Author**

Reviewer #1: All comments have been addressed

Reviewer #2: All comments have been addressed

**********

2. Is the manuscript technically sound, and do the data support the conclusions?

Reviewer #1: Yes

Reviewer #2: Yes

**********

3. Has the statistical analysis been performed appropriately and rigorously?

Reviewer #1: N/A

Reviewer #2: Yes

**********

4. Have the authors made all data underlying the findings in their manuscript fully available?

The PLOS Data policy

Reviewer #1: Yes

Reviewer #2: (No Response)

**********

5. Is the manuscript presented in an intelligible fashion and written in standard English?

Reviewer #1: Yes

Reviewer #2: Yes

**********

Reviewer #1: (No Response)

Reviewer #2: I would like to thank the authors for carefully addressing all the raised comments. In my opinion, the manuscript is now ready for acceptance and publication in the PLOS One journal.

**********

what does this mean?). If published, this will include your full peer review and any attached files.

If you choose “no”, your identity will remain anonymous but your review may still be made public.

**Do you want your identity to be public for this peer review?**  For information about this choice, including consent withdrawal, please see our Privacy Policy

Reviewer #1: No

Reviewer #2: No

**********

# Editor report: Acceptance letter

**DOI:** 10.1371/journal.pone.0358863.r004

**Authors:** Rohan Kothurkar

PONE-D-26-04622R1

PLOS One

Dear Dr. Conconi,

I'm pleased to inform you that your manuscript has been deemed suitable for publication in PLOS One. Congratulations! Your manuscript is now being handed over to our production team.

At this stage, our production department will prepare your paper for publication. This includes ensuring the following:

* All references, tables, and figures are properly cited

* All relevant supporting information is included in the manuscript submission,

* There are no issues that prevent the paper from being properly typeset

You will receive further instructions from the production team, including instructions on how to review your proof when it is ready. Please keep in mind that we are working through a large volume of accepted articles, so please give us a few days to review your paper and let you know the next and final steps.

Lastly, if your institution or institutions have a press office, please let them know about your upcoming paper now to help maximize its impact. If they'll be preparing press materials, please inform our press team within the next 48 hours. Your manuscript will remain under strict press embargo until 2 pm Eastern Time on the date of publication. For more information, please contact onepress@plos.org.

You will receive an invoice from PLOS for your publication fee after your manuscript has reached the completed accept phase. If you receive an email requesting payment before acceptance or for any other service, this may be a phishing scheme. Learn how to identify phishing emails and protect your accounts at https://explore.plos.org/phishing.

If we can help with anything else, please email us at customercare@plos.org.

Thank you for submitting your work to PLOS One and supporting open access.

Kind regards,

PLOS One Editorial Office Staff

on behalf of

Dr. Rohan Kothurkar

Academic Editor

PLOS One
